# Novel dihydroxybenzohydrazide grafted deoxycellulose for efficient removal of anionic food colorants and hexavalent chromium from wastewater

**DOI:** 10.1038/s41598-025-14609-5

**Published:** 2025-08-13

**Authors:** Magda A. Akl, Azza A. H. Fahim, El-Sayed R. H. El-Gharkawy

**Affiliations:** Department of Chemistry, Faculty of Science, Mansoura University, Mansoura, 31556 Egypt

**Keywords:** Deoxycellulose, Adsorption, Cr(VI), Food dyes, DFT calculations, Wastewater, Infrared spectroscopy, Chemistry, Analytical chemistry, Environmental chemistry, Materials chemistry, Polymer chemistry, Theoretical chemistry

## Abstract

In this study, 2,4-dihydroxybenzaldehyde hydrazone (CELL@HBH) adsorbent was prepared from microcrystalline cellulose powder (CELL) through a simple and effective method. The investigated materials were characterized by Fourier Transform Infrared spectroscopy (FT-IR), Scanning electron microscopy (SEM), elemental analysis, Proton Nuclear Magnetic Resonance (^1^HNMR), Thermogravimetric analysis (TGA), X-ray diffraction (XRD), and Brunauer-Emmett-Teller (BET) surface area measurements. The prepared (CELL@HBH) adsorbent was applied to remove anionic food colorants carmoisine (E122), ponceau 4R (E124), and Cr(VI) from polluted water samples. The effects of pH, initial concentration, contact time, dosage, temperature, and competing ions have been investigated to maximize the adsorption capacities to reach 476.709 mg/g, 338.789 mg/g, and 190.072 mg/g for E122, E124, and Cr(VI) ions, respectively, at the optimum conditions. The results showed that the adsorption of E122, E124, and Cr(VI) follows pseudo-2nd-order kinetic and Langmuir isotherm models due to the more subordinate error functions and increased correlation coefficient (R^2^ ≥ 0.999). Thermodynamic studies indicate the adsorption of E122 and E124, as well as Cr(VI) ions, by CELL@HBH adsorbent to be exothermic and spontaneous. DFT calculations were employed to verify the molecular structure, analysis of Frontier Molecular Orbitals (FMOs), molecular electrostatic potential (MEP), and geometry reactivity descriptors (GRDs) for all phases. The prepared adsorbent effectively removed E122, E124, and Cr(VI) from polluted water samples, synthetic mixtures, and colored soft drinks, with a recovery rate of ~ 97%. The CELL@HBH adsorbent has good recycling performance. Under five regeneration and adsorption cycles, it still has removal effect greater than 85% of E122, E124, and Cr(VI), which indicates its high structural stability. The adsorption mechanism of E122, E124, and Cr(VI) onto CELL@HBH adsorbent is elucidated. Ultimately, this study demonstrates that the fast-responsive CELL@HBH adsorbent can be effectively utilized to eliminate E122, E124, and Cr(VI) from a wide range of real water sources. Collectively, the results indicate that the as-prepared CELL@HBH adsorbent is promising for anionic pollutant adsorption and our mechanistic results are of guiding significance in environmental cleanup. This work contributes significantly to understanding how experimental conditions influence the mechanism of E122, E124, and Cr(VI) adsorption by CELL@HBH adsorbent, offering valuable and new insights for future applications and optimizations in the treatment of effluent-containing anionic species.

**Supplementary Information:**

The online version contains supplementary material available at 10.1038/s41598-025-14609-5.

## Introduction

The availability of safe water is increasingly scarce in numerous regions of the world^1^. Many people all over the world struggle to get enough water to drink because of the agricultural needs, manufacturing demands, and population expansion^2^. In developing nations, about 6 million children per year die as a result of the water pollution problem, according to the WHO. Water can be polluted by organic matters such as herbicides, fuel, synthetic dyes, and hydrocarbons, in addition to inorganic ones, such as salts, acids, phosphates, sulfates, and hazardous heavy metals^3^. Synthetic dyes are classified in the category of organic chemical pollution. Dyes can be classified into three main classes (cationic, anionic, or non-ionic)^4^.

Anthraquinone, azo chromophores, are present in many anionic dyes^5^. Approximately 65% of azo dyes are utilized as food additives in various products, including soft drinks, jams, sweets, and pickles^6^. The beverage industry’s extensive use of artificial food dyes has immensely improved the aesthetic appeal of various consumables. Although there is an urgent need for sustainable and efficient ways to lessen these dyes’ negative effects on the environment and human health, particularly in children, as a result of strict environmental rules and growing awareness about the possible health concerns they pose^7^. Carmoisine (E122) and Ponceau 4R (E124) are common food anionic azo dyes^8^. Significant amounts of them end up in the water effluents, which increases the threat to our lives. The main danger may be due to the cleavage of the azo bond and the formation of carcinogenic aromatic amines. These amines are formed either by the assistance of intestinal microorganisms or due to the presence of azo reductase enzyme in the liver or intestinal wall^9^. Moreover, the generated aromatic amines in the colon and the liver are eliminated through the kidney^10^. Sulfanilic acid, which is produced in this reaction as a key product, has the ability to influence cell division, hence leading to carcinogenesis^11^. Food dyes are nonbiodegradable because of their physicochemical, thermal, and optical stability resulting from their symmetrical aromatic groups. So, their elimination is essential in order to overcome their adverse effects^12^. A value of 0–4 mg/kg body weight is the acceptable daily intake of E122 and E124 according to the WHO^7^.

Heavy metals are also classified as dangerous contaminants that pose a great threat to human health and the environment. Chromium (Cr), with two forms: hexavalent [Cr(VI)] and trivalent [Cr(III)], is considered to be one of these dangerous heavy metals. As Cr(III) is insoluble and primarily bonded to organic matter, it is less dangerous than Cr(VI), which is highly soluble, movable, and toxic^13^. According to the WHO guidelines, the limited Cr(VI)^−^ in drinking water is ≤ 0.05 mg/L^14^.

Recently, numerous methods have been developed to reduce the levels of pollution in the wastewater such as membrane systems^15^, flotation^16^, adsorption^17^, chemical precipitation^18^, and ion exchange^19^.

From an economic and environmental standpoint of view, adsorption has gained favor over these traditional methods. Adsorption is a powerful technique for removing anionic, cationic, or organic contaminants from one phase and transporting them to the outside surface via complexation or ionic exchange and has numerous advantages for wastewater treatment, including inexpensiveness, flexibility, design simplicity, economic viability, effectiveness, and sensitivity to harmful contaminants without the production of secondary pollutants^20^. Many adsorbents for contaminants’ removal have been documented, including activated carbon^21^, clays^22^, chitosan^23^, cellulose and biomass^24^, as well as other compounds such as resin^25^.

Cellulose is an adsorbent that has a large potential for eliminating pollutants from wastewater because of its traditional nature, low cost, and high efficiency, remarkable adsorption capacities, unique structural features, renewable nature, availability, ability to regenerate, environmental friendliness, and biodegradability^26^.

The purpose of cellulose modifications is to add functional groups. The three hydroxyl groups that are present in every cellulose unit allow cellulose to be readily modified. Numerous methods, including oxidation^27^, esterification^28^, etherification^29^, and halogenation, are used to modify cellulose.

One of the most well-known ways for modifying cellulose is to convert the major C-6 hydroxyl groups to excellent leaving groups like tosylate, mesylate, chloride, bromide, or iodide, and then carrying out a nucleophilic displacement reaction. A variety of cellulose derivatives containing azides, amines, thiols, and heterocycles have been made using this method^30,31^.

Various adsorbents have been applied for the adsorption of the E122 and E124 food dyes. It was observed that some limitations in these investigations were that (i) the majority of these adsorbents were utilized for the removal of these dyes in a single system, (ii) most of the applied adsorbents were high-cost, low adsorption efficiency, and low reusing ability, and (iii) cellulose-based adsorbents presents an ecofriendly, low cost, available adsorbent but suffer from low adsorption capacity toward anionic species so need further modifications^32,33^. Consequently, there is a vital need for preparing an adsorbent with the following characteristics: reusable, high adsorption capability, cost-effective, and effectively applied in single and multiple systems in various water samples. Mostafa et al. prepared the bentonite modified with CTAB for the removal of E122 and E124 in single and binary systems^12^. Kieu et al. used zeolite modified with surfactant for the E124 removal^34^. Moreover, Zhu et al. applied the alkali-treated fish scales for the E124 adsorption^35^.

Various published investigations have utilized cellulose-modified adsorbents for the efficient removal of various pollutants. The cellulose modification can be carried out to achieve enhanced physical and chemical properties. As the introduction of polyethyleneimine to natural cellulose resulted in better adsorption of mercury^36^, as well as of Cr^3+^and Fe^3+^^37^ than unmodified cellulose. Also, cellulose modified with glycidyl methacrylate and functionalized by imidazole has revealed significant removal of Pb^2+^ from aqueous solutions^38^. Complexation between cellulose and ethylene-diamine-tetra-acetic dianhydride has also enhanced the adsorption behavior of cellulose towards Cu^2+^, Cd^2+^, and Pb^2+^ ions^39^. Also, the functionalized Flax fibers with the semicarbazide were utilized to eliminate both Cr(VI) and Alizarin Red S dye from various water samples^12^. Mostafa et al. modified the dialdehyde cellulose by utilizing cyanoacetohydrazide and CS_2_, respectively, and used the modified material for the Hg(II) selective removal^40^.

The novelty of the current investigation lies in the preparation of a novel, affordable, eco-friendly, and reusable adsorbent that has a high efficiency toward anionic species. Moreover, it has high stability in various pH media and at different temperature degrees. According to our knowledge, no research has been reported on the following points:- (i) The formation of CELL@HBH adsorbent through the modification of chlorinated cellulose with di-hydroxybenzaldehyde ligand as well as its characterization, (ii) the use of CELL@HBH for the effective removal of E122, E124, and Cr(VI) from different polluted water samples in single and mixed systems and (iii) studying the molecular structure, electronic properties, and the quantum mechanical calculations of CELL, CELL@HBH, CELL@HBH@E122, CELL@HBH@E124, and CELL@HBH@Cr(VI).

The objectives of the present study are presented in the following points: (i) Modification of native cellulose (CELL) to create CELL@HBH adsorbent; (ii) Characterization of CELL and CELL@HBH using different instrumental performances as elemental analysis, SEM, BET, FTIR, ^1^HNMR, XRD and TGA; (iii) Experimental investigation of anionic pollutants E122, E124, and Cr(VI) adsorption on the surface of CELL@HBH; (iv) Studying the adsorption of E122, E124, and Cr(VI) anionic species in in multi-contaminant systems; (v) Investigating the different experimental parameters affecting the adsorption process of E122, E124, and Cr(VI) onto CELL@HBH adsorbent, e.g., adsorbent dosage, pH, initial of E122, E124, and Cr(VI) concentrations, and ionic strength; (vi) Conducting adsorption isotherm and kinetic experiments to understand the adsorption mechanism and highest adsorption ability of CELL@HBH adsorbent; (vii) Application of the prepared CELL@HBH adsorbent on real water and soft drinks samples; (viii) Elucidation of the adsorption mechanism of E122, E124, and Cr(VI) onto CELL@HBH adsorbent.

## Experimental

### Materials

Cellulose powder, phosphorus oxychloride (POCl_3_), dimethyl formamide (DMF), NaOH (5%), Acetic Acid (5%), Hydrazine Hydrate (HH), Ethanol, Carmoisine dye (C_20_H_12_N_2_Na_2_O_7_S_2_, E122), Ponceau 4R dye (C_20_H_11_N_2_Na_3_O_10_S_3_, E124), Cr(VI), 2,4 dihydroxy benzaldehyde (DHB) and HCl were all analytical grade and they were purchased from Merk.

### Preparations

#### Preparation of deoxycellulose chloride (CELL-Cl)

A sample of 1 g of cellulose powder was suspended in 20 mL of dimethyl formamide (DMF) for about 90 min. After that, 1 mL of phosphorus oxychloride (POCl_3_) was added to the reaction under mechanical stirring in a water bath at 90 °C for about 1 h. The brown product, obtained from this chlorination step, was filtered off, then washed with DMF, distilled water, and NaOH (5%), and finally with 5% acetic acid solution followed by distilled water. The CELL-Cl became white again after these washing steps, then dried in air for 24 h^41^.

#### Preparation of deoxy-cellulose hydrazide (CELL@HH) and estimation of the chloro group content

Firstly, a sample of 0.5 g CELL-Cl was directly reacted with 5 mL of HH without using any solvent. The reaction mixture was stirred under heating for 3 h at 80 °C. The product (CELL-HH) obtained was filtered off, washed with DDW, followed by ethanol, and then dried in air.

In the second step, the concentration of the amino group of (CELL-HH) was estimated using a volumetric method as follows: 40 mL of 0.05 M HCl solution was added to 0.1 g of (CELL-HH) and conditioned for 15 h on a Vibromatic Shaker. The residual concentration of HCl was estimated through the titration against 0.05 M NaOH solution and phenolphthalein as an indicator. The number of moles of HCl interacted with amine groups and consequently the amine group concentration (mmol/g) was calculated from the following Eq. (1).

1 $$\:Concentration\:of\:amino\:groups=\frac{({M}_{1}-{M}_{2})}{0.1}\times\:40\left(\frac{mmole}{g}of\:adsorbent\right)$$

where, M_1_ and M_2_ are the initial and final concentrations of HCl, respectively^42^.

#### Preparation of 2,4 di-hydroxy benzaldehyde hydrazone supported deoxycellulose (CELL@HBH) adsorbent

**Fig. 1:**
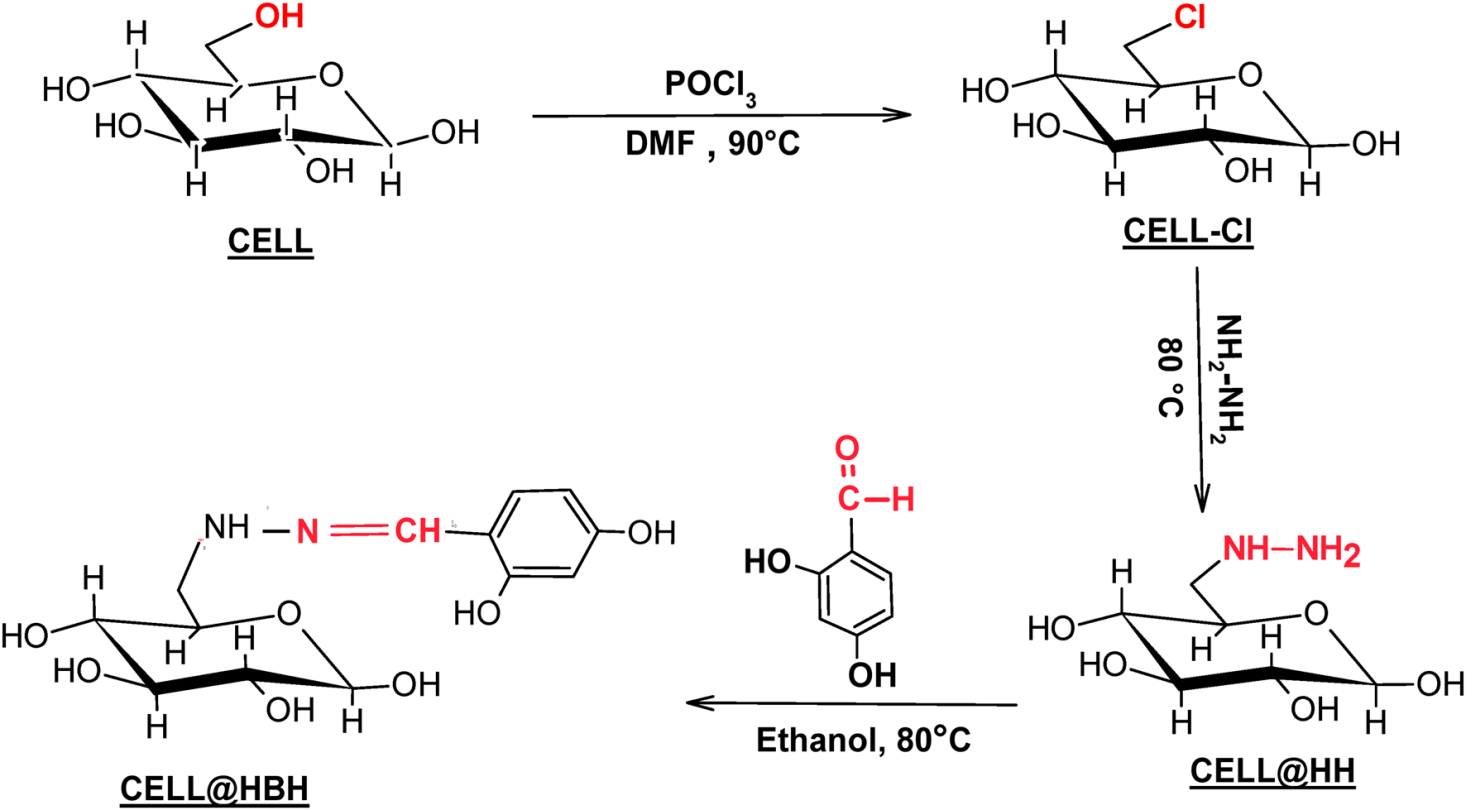
Preparation of CELL @HBH.

0.3 g of the CELL@HH was added to 0.1 g of the 2,4-dihydroxybenzaldehyde (DHB) in the presence of 20 mL of ethanol solvent. The reaction mixture was stirred in reflux at 80 °C for 6 h. After filtering out the desired material, it was washed with absolute ethanol, then with hot distilled water. And finally, after the washing steps, it was dried in air. The synthetic steps of CELL-HBH are illustrated in Fig. 1.

**Fig. 2:**
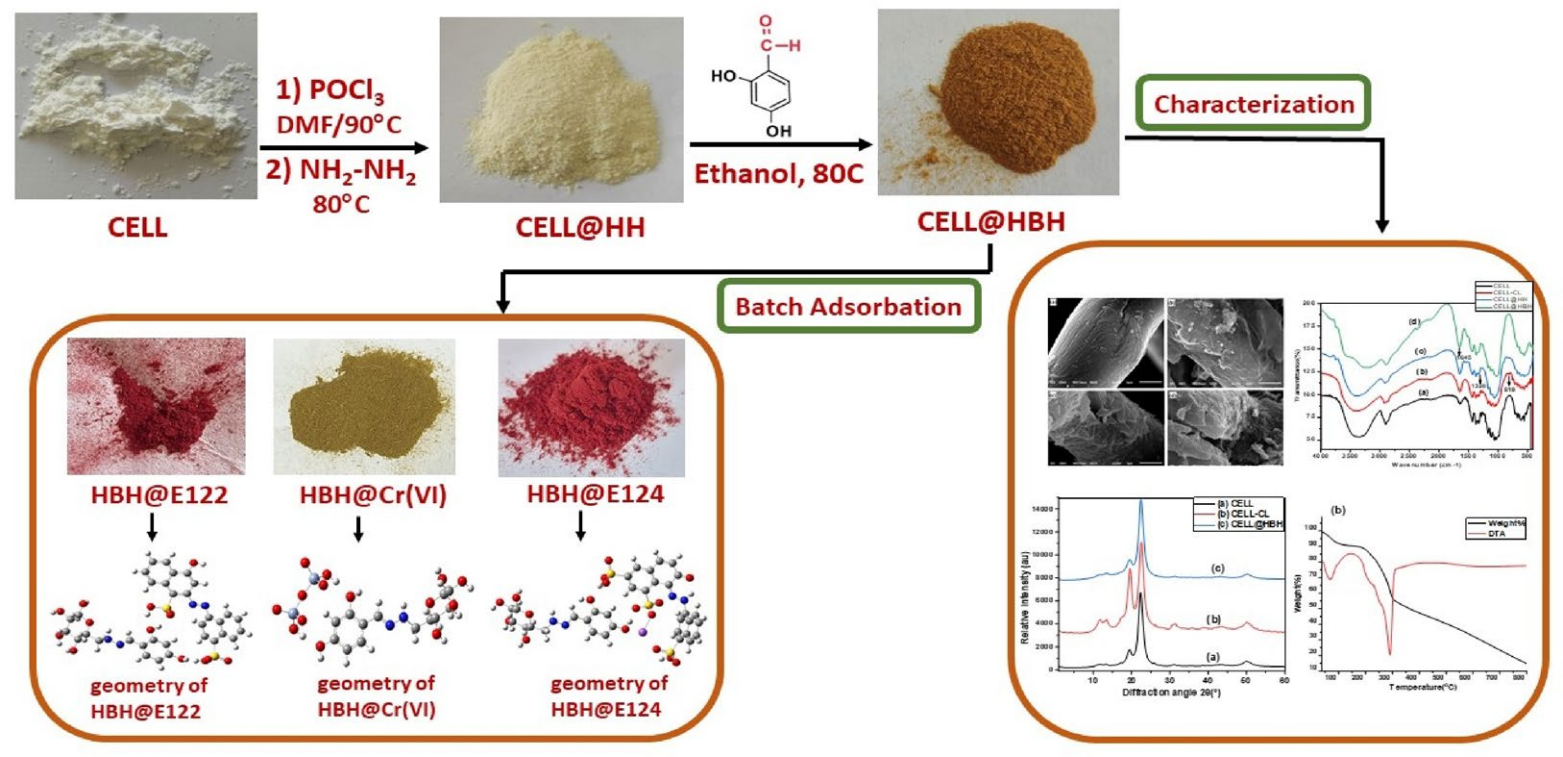
Synthesis and characterization of CELL@HBH and its usage for E122, E124, and Cr(VI) adsorption.

The synthesis and characterization of CELL@HBH adsorbent and its use for the removal of E122, E124, and Cr(VI) are graphically represented in Fig. 2.

###  Instrumentation

Fourier transform infrared (FT-IR) spectra and the Surface morphologies (SEM) of the prepared materials were recorded using a Perkin-ElmerSpectrum RX Iusing KBr pellets and Scanning Electron microscopy (A JSM-6510LV), respectively. A PAN analytical X’Pert PRO diffractometer was employed for measuring the X-ray diffraction (XRD) patterns of the CELL, CELL-Cl, and CELL@HBH samples at (4–70°) range of 2-theta (2θ). The specific surface area (S_**BET**_) of CELL, CELL-Cl, and CELL@HBH materials was investigated at a temperature of 77 K utilizing the Brunauer Emmet Teller (BET) analysis (Size Analyzer (QUANTACHROME—NOVA 2000 Series). Prior to the BET analysis to remove any moisture, 100 mg of the sample was degassed at a temperature of 120 ^ο^C under a 10^− 3^ mbar vacuum for 12 h. The S_BET_ was calculated as presented in Fig. S1, corresponding to the N_2_ adsorption isotherm. The CHNO composition determination of the prepared materials was obtained by a Costech ECS-4010 elemental analyzer. The thermal stability of CELL-Cl and CELL@HBH materials was examined by thermogravimetric analysis (Berkin Elmer TGA 4000) at a heating rate of 15 °C/min from 30 to 900 °C. The ^1^HNMR spectra of the prepared materials were measured in a mixture of DMSO/trifluoroacetic acid (TFA) using a Joel 500 MHZ Japan.

In order to determine the pH_PZC_, 10 mL of a 0.1 M NaCl solution was added in a series of glasses. 0.1 M HCl or 0.1 M NaOH was added to create solutions with an initial pH (pH_i_) ranging from 2 to 12. After that, 0.01 g of adsorbent were added to each solution, and they were all stirred for 48 h. The final pH value (pH_f_) was obtained, and the difference between the pH values at the beginning and the end (ΔpH = pH_i_-pH_f_) was plotted against pH_i_. The value of pH_pzc_ is the point at which the ΔpH vs pH_i_ curve crosses the ΔpH = 0 line.

### Adsorption studies

####  Batch adsorption

The adsorption behavior of E122, E124, and Cr(VI) using CELL@HBH adsorbent was investigated through batch adsorption experiments in 20 mL of each of them and a 0.0075 g of CELL@HBH adsorbent dose. Various parameters were investigated including pH from 2.0 to 12.0 for both E122 and E124 and from 2.0 to 8.0 for Cr(VI) ions, analyte concentration of (50–300 mg/L) for E122, Cr(VI) and (100–300 mg/L) for E124, adsorbent dose (0.005–0.02 g), and contact time (15–180 min) for E122 and E124 and (120 to 360 min) for Cr(VI). The pH was adjusted using diluted HCl (0.1 M) and NaOH (0.1 M) solutions. The removal (%) and adsorption capacity at equilibrium (q_e_) were determined using Eqs. (2) and (3), respectively.

2 $$\:\text{R}\left(\text{\%}\right)=\frac{{\text{C}}_{\text{i}}-{\text{C}}_{\text{f}}}{{\text{C}}_{\text{f}}}\times\:\:100$$

3 $$\:{\:\:\text{q}}_{\text{e}}=\frac{({\text{C}}_{\text{i}}-{\text{C}}_{\text{f}})\times\:\text{v}}{\text{m}}$$

C_i_ and C_f_ are the initial and equilibrium concentration (mg/L), respectively. While m (g) is the CELL@ HBH dose and V (L) is the adsorbate solution volume.

##### Effect of initial concentration and isotherm investigation

In order to evaluate the influence of initial concentration and investigate the isotherm studies, 20 mL solution of E122, E124, and Cr(VI) were added to a 0.0075 g of CELL@HBH adsorbent at pH of 2 and subjected to a thermostatic shaker at 150 rpm for 2 h for the two dyes and 5 h for Cr(VI) at a temperature of 25 °C.

Three isotherm models were studied, namely the Langmuir, Freundlich, and Dubinin–Radushkevich (D-R) models. The Langmuir isotherm postulates that once an adsorbate has occupied a spot, no more adsorption occurs there, creating a discriminating plateau in the curve, and there is no side commerce or steric interference between molecules that have been adsorbed^43^. The model equation and the Langmuir separation factor (R_L_), which is an important parameter that is used to calculate affinities between adsorbents and sorbates, are represented by Eqs. (4) and (5).

On the other hand, the Freundlich isotherm could be considered an empirical model in which there is an interaction between adsorbed molecules (multilayer adsorption) on heterogeneous surfaces with a uniform energy distribution. Also, this model proved that the adsorbate concentration on the adsorbent surface will be increased if there is a growth of adsorbate concentration in the solution without attaining saturation^44^. The Freundlich isotherm model is represented by Eq. (6). The D–R isotherm model assumes that biosorption is related to surface porosity and pore volume, and examines biosorption energetically. The mean free energy of biosorption (E_DR_) obtained from the D–R model determines whether the adsorption structure is chemical or physical. 8 < E_DR_ < 16 kJ mol^− 1^ indicates that the adsorption has a chemical character. If E_DR_ < 8 kJ mol^− 1^, it determines that the adsorption is physical. The model equation is represented by Eq. (7).

4 $$\:\frac{{C}_{e}}{{q}_{e}}=\:\:\frac{1}{{K}_{L}{q}_{m}}+\frac{{C}_{e}}{{q}_{m}}\:$$

5 $$\:{\text{R}}_{\text{L}\:}=\frac{1}{1+{K}_{L}{C}_{0}}$$

6 $$\:\text{l}\text{n}\:{\text{q}}_{\text{e}}=\:\text{l}\text{n}{\text{k}}_{\text{f}\:}+\frac{1}{\text{n}}\text{l}\text{n}\:{\text{C}}_{\text{e}}$$

7 $${\text{ln }}{{\text{q}}_{\text{e}}} = {\text{ ln }}{{\text{q}}_{\text{m}}} - {\text{ k}}{\varepsilon ^{\text{2}}}$$

Where, C_e_ (ppm) is the initial concentration of the studied pollutant at equilibrium, q_e_ (mg/g) is the capacity of the adsorbent for pollutant concentration at equilibrium, q_m_ (mg/g) adsorption maximum amount, 1/n, K_L_, K_F_, and K_DR_ are the heterogeneity factor, Langmuir coefficient (L/mg), Freundlich constant ((mg^1−(1/n)^⋅L^1/n^)/g), and the Dubinin–Radushkevich constant, respectively. While ε is the adsorption potential and is given by Eq. (8).

8 $$\:{\upepsilon\:}=\text{R}\text{T}\text{l}\text{n}\left.\left(1\:+\:\frac{1}{{C}_{e}\:}\right.\right)$$

where, R (8.314 J/mol K) is the gas constant, and T is the temperature in kelvin.

##### Effect of contact time and kinetic studies

20 mL solution of 200 mg/L E122, 150 mg/L E124 and100 mg/L Cr(VI) were added to 0.0075 g of CELL@HBH adsorbent at pH 2 and subjected to a thermostatic shaker at 150 rpm at a temperature of 25 °C in order to evaluate the equilibrium time for the adsorption process and to investigate the kinetic studies.

The adsorption process is actually controlled by the slowest step, as the liquid solution is being adsorbed onto the solid adsorbent surfaces in multiple phases, so Pseudo-1st -order, pseudo-2nd -order, and intraparticle diffusion (IPD) kinetic models were studied^45^.

Pseudo-1st -order, pseudo-2nd -order, and IPD models are expressed in Eqs. (9), (10) and (11).

9 $$\:\frac{1}{{\text{q}}_{\text{t}}}=\:\frac{{\text{K}}_{1}}{{\text{q}}_{\text{e}}}+\frac{1}{{\text{q}}_{\text{e}}}$$

10 $$\:\frac{\text{t}}{{\text{q}}_{\text{t}}}=\:\frac{1}{{\text{K}}_{2}{\text{q}}_{\text{e}}^{2}}+\frac{1}{{\text{q}}_{\text{e}\:}\text{t}}\:$$

11 $$\:{\text{q}}_{\text{t}}={\text{K}}_{\text{d}\text{i}\text{f}\text{f}}\:{\times\:\text{t}}^{1/2}+\text{C}$$

Where, q_e_ (mg/g) and q_t_ (mg/g) are the adsorption efficiency at equilibrium and at a specific time t (min), respectively. C is the intercept that reflects the boundary layer effect. Further, K_1_, K_2_, and K_diff_ are pseudo-1st, pseudo-2nd order, and intra-particle diffusion constants, respectively.

##### Effect of temperature and thermodynamic studies

20 mL solution of 200 mg/L E122, 150 mg/L E124 and100 mg/L Cr(VI) were added to 0.0075 g of CELL@HBH adsorbent at pH 2 and subjected to a thermostatic shaker at 150 rpm at different temperatures between 30 and 45 °C in order to evaluate the equilibrium time for the adsorption process in and to investigate the kinetic studies.

In order to better understand the effect of rising temperature on the adsorption of the three pollutants onto the active sites of the CELL@HBH, three basic thermodynamic parameters were studied: the Gibbs free energy of adsorption ($\:{{\Delta\:}\:\text{G}}_{\text{a}\text{d}\text{s}}^{\text{o}}$), the enthalpy change ($\:{{\Delta\:}\:\text{H}}_{\text{a}\text{d}\text{s}}^{\text{o}}$), and the entropy change ($\:{{\Delta\:}\:\text{S}}_{\text{a}\text{d}\text{s}}^{\text{o}}$)^46^. These parameters for the adsorption process were determined by using Eqs. (12), (13), and (14).

12 $${\text{K}}_{\text{c}}=\frac{{\text{C}}_{\text{a}\text{d}}}{{\text{C}}_{\text{e}}}$$

13 $${{\Delta\:}\:\text{G}}_{\text{a}\text{d}\text{s}}^{\text{o}}=\:-\text{R}\text{T}\text{I}\text{n}\:{\text{K}}_{\text{C}}$$

14 $$\text{l}\text{n}\:{\text{K}}_{\text{C}}=\:\frac{{{\Delta\:}\:\text{S}}_{\text{a}\text{d}\text{s}}^{\text{o}}}{\text{R}}-\:\frac{{{\Delta\:}\:\text{H}}_{\text{a}\text{d}\text{s}}^{\text{o}}}{\text{R}\text{T}}$$

where K_c_ is the thermodynamic equilibrium constant, but C_ad_ and C_e_ are the pollutant concentration taken by the adsorbent material at equilibrium (mg/g) and the pollutant concentration at equilibrium (mg/L), respectively. While R represents the universal gas constant, that is equivalent to 8.314 J/mol K.

##### Removal of E122, E124, and Cr(VI) in multi-contaminant systems

To examine the effectiveness of CELL@HBH in multi-contaminant systems of E122, E124, and Cr(VI), 0.0075 g of CELL@HBH were placed in 20 mL of each binary system solution containing 100 mg/L of each pollutant at pH 2 for the E122 + E124 combination and pH 4 for the other combinations, as Cr(VI) present in K_2_Cr_2_O_7_ solution worked as an inorganic oxidizing agent in very acidic medium and caused deceleration of E122 and E124 azo dyes^47^. The mixtures were then subjected to shaking at 120 rpm, and throughout a 4 h period, the maximum adsorption for each binary system was recorded hourly.

### Error functional analysis

In order to analyze the error distribution between calculated values based on theoretical model correlations and experimental data, a variety of error functions were used to evaluate the isotherm models. The chi-square statistic (χ2), mean square error (MSE), and sum of squares error (SSE), which are explained in Eqs. (15), (16), and (17) respectively, were the three error functions that were utilized^48^.

15 $$\:{\chi\:}^{2}=\sum\:_{i=1}^{n}\frac{({{q}_{ei\:}exp-{q}_{ei}\:cal)}^{2}}{{q}_{ei}\:cal}$$

16 $$\:\:\:\text{M}\text{S}\text{E}=\frac{1}{{N}_{exp}}{\sum\:}_{i=1}^{n}({{q}_{ei\:}exp-{q}_{ei}\:cal)}^{2}$$

17 $$\:\text{S}\text{S}\text{E}={\sum\:}_{i=1}^{n}({{q}_{ei\:}exp-{q}_{ei}\:cal)}^{2}$$

where, n is the number of included observations. The subscript cal refers to theoretically calculated data while exp subscript represent experimental data.

### Desorption and regeneration

The batch technique was employed to study the regeneration of CELL@HBH over five repeated cycles of the adsorption–desorption procedure. Adsorption experiments for Cr(VI), E122 and E124 were carried out by adding 0.0075 g of CELL@HBH adsorbent to 20 mL solutions of 200 mg/L E122, 200 mg/L E124 and 100 mg/L Cr(VI) for 2 h for both E122 and E124 and 5 h for Cr(VI) ions. After that, CELL@HBH was filtered and washed. For the investigation of the desorption process, 0.0075 g of CELL@HBH@E122, CELL@HBH@E124 were added to 20 mL EDTA (0.1 mol/L) and 0.0075 g of CELL@HBH@Cr(VI) in NaOH (0.5 M); then shaking for 2 h For CELL@HBH@E122, CELL@HBH@E124 and 5 h for CELL@HBH@Cr(VI). This process was repeated for five times. The desorption efficiency is the ratio between the amount desorbed to the solution to the amount adsorbed by CELL@HBH^49^.

The Desorption efficiency can be calculated using Eq. (18).

18 $$\:\text{D}\text{e}\text{s}\text{o}\text{r}\text{p}\text{t}\text{i}\text{o}\text{n}\%=\:\frac{\text{a}\text{m}\text{o}\text{u}\text{n}\text{t}\:\text{d}\text{e}\text{s}\text{o}\text{r}\text{b}\text{e}\text{d}\:\text{t}\text{o}\:\text{t}\text{h}\text{e}\:\text{s}\text{o}\text{l}\text{u}\text{t}\text{i}\text{o}\text{n}\:(\text{m}\text{g}.\:{\text{g}}^{-1})}{\text{a}\text{m}\text{o}\text{u}\text{n}\text{t}\:\text{a}\text{d}\text{s}\text{o}\text{r}\text{b}\text{e}\text{d}\:\text{b}\text{y}\:\text{C}\text{E}\text{L}\text{L}@\text{H}\text{B}\text{H}\:\:(\text{m}\text{g}.\:{\text{g}}^{-1})}x100$$

### Application

#### In natural water samples

To investigate the applicability of CELL@HBH in removal of studied pollutants from real water samples (Tap, waste and seawater). Samples were spiked with 50, 100, 150, and 200 of E122, E124, and Cr(VI). In advance of the spiking of the pollutants, the natural water samples were digested by the addition of 0.5 g of K_**2**_S_**2**_O_**8**_ and 5 mL H_**2**_SO_**4**_ 98% (w/w) to 1000 mL of water sample and heated for 120 min at 90 °C for complete digestion of organic materials^50^. Following the samples’ room temperature cooling, 0.0075 g of CELL@HBH was added. The samples were then continuously shaken for 120 min for E122, E124, and 300 min for Cr(VI) at pH 2. Using the appropriate wavelengths (518 nm for E122, 508 nm for E124, and 427 nm for Cr(VI)), a Unicam UV 2100 UV/Visible spectrometer was used to determine the remaining amounts of E122, E124, and Cr(VI).

#### In colored drinks and industrial samples

CELL@HBH was used to extract E122 from degassed carbonated soft drinks, gum, as well as extraction of E124 from jelly. The carbonated soft drinks (flavored with pomegranate and dyed with E122) were first degassed by exposing them to the air at 25 °C for 120 min. Following digestion (i.e., the breakdown of undesirable substances in the sample) with 4% acetic acid, the E124 dye, strawberry flavoured, gum and jelly were dissolved in distilled water^51,52^.

0.0075 g of CELL@HBH adsorbent were added to each sample for E122 and E124, and continuous shaking was used for 120 min at pH 2 for each dye. The remaining E122 and E124 were determined using the Unicam UV 2100 UV/Visible spectrometer at the appropriate wavelengths (518 nm for E122, 508 nm for E124, and 427 nm for Cr(VI)).

### DFT calculations

Geometry optimizations and other DFT calculations were performed on the CELL, HBH, CELL@HBH, E122, E124, Cr(VI), CELL@HBH@E122, CELL@HBH@E124 and CELL@HBH@Cr(VI). DFT is considered a cost-effective method to approximate electron correlation effects. All DFT calculations were performed by using B3LYP level of theory, Becke’s three parameter (B3) nonlocal exchange with the correlation functional of Lee, Yang, and Parr (LYP)^53^. Nowadays, the B3LYP level is currently widely used to study organic electronic compounds because the predicted geometries are very reliably and provides good estimations for HOMO–LUMO gaps, in a good agreement with experimental values^54–57^. All the calculations, for geometry optimizations were carried out at the B3LYP/6–31 g (d, p) for all atoms. All computations were carried out by using Gaussian09 suite of program^58^. and full natural bond orbital (NBO) analyses were made to calculate the charge distribution for all molecules by using NBO version 3.1^59^. Gauss View 5.0 package^60^ was used to obtain various graphic views of molecular shapes of distinctive molecular orbitals.

Highest Occupied Molecular Orbitals (HOMO), and Lowest Unoccupied Molecular Orbitals (LUMO) are very considerable elements of theoretical molecular design^61^. The electronic properties and reactivity definers such as ionization potential (I_P_), electron affinity (E_A_), hardness ($\:\eta\:)$, softness ($\:\sigma\:$), electronegativity ($\:\chi\:$), chemical potential $\:\left(\mu\:\right)$, and electrophilicity index (ω) can be determined from the HOMO and LUMO orbital energies^62–66^ through Koopman’s theorem^67^. Equations used to calculate these reactivity descriptors are presented in Table S1.

## Results and discussion

### Characterization

#### Elemental analysis

##### Cl-content analysis

Since modification of cellulose occurred at Cl atom, it is necessary to calculate the Cl content in CELL-Cl. This was carried out by substituting Cl with NH_2_ group, then using the volumetric method to estimate the NH_2_ content which is equivalent to Cl content. The analysis revealed that CELL-Cl contains 3.34 mmol-Cl/g).

##### CHNO analysis of materials

The elemental analysis was evaluated for CELL, CELL-Cl, CELL@HH and CELL@HBH materials to prove the modification steps. The results obtained in Table 1 indicate that the carbon, oxygen and hydrogen content decreased from 46.51%, 46.46%, and 7.03–42.65%, 44.19% and 6.16%, respectively and this may be due to the modification that occurred through the cellulose chlorination.

In the second step, nitrogen content of CELL@HH became 5.58% due to the hydrazine groups introduced instead of chlorine atoms.

In final step of modification, carbon content increased to 48.23% as a result of the insertion of ligand with an aromatic ring rich with carbon atoms to form CELL-HBH. These results confirm that CELL-Cl, CELL@HH and CELL@HBH are successfully formed.

**Table 1:** Elemental analysis.

Material	C%	H%	O%	*N*%
CELL	46.51	7.03	46.46	0
CELL-Cl	42.65	6.16	44.19	0
CELL@HH	43.03	6.82	44.58	5.58
CELL@HBH	48.23	6.15	41.12	4.50

#### N_2_ adsorption/desorption isotherm Brunauer–Emmett–Teller (BET) analysis

Using the BET method, the surface area of CELL-Cl and CELL@HBH was examined (Fig. S1) and the findings, which are displayed in Table S2, demonstrate that the BET surface area dropped from 41.007 to 0.390 m^2^/g for CELL and CELL@HBH respectively. The significant decrease in the surface area after the modification step with the DHB ligand may be due to numerous reasons, like: (i) the occupation of the surface pores by the ligand particles, which lowers the adsorption of N_2_ molecules used in the surface area measurement process^12^, (ii) the coating of the surface with the ligand, leading to a decrease in the surface roughness^68^, and (iii) the reduction of external surface area due to the partial agglomeration of the cellulose^69^.

#### FTIR spectra

**Fig. 3:**
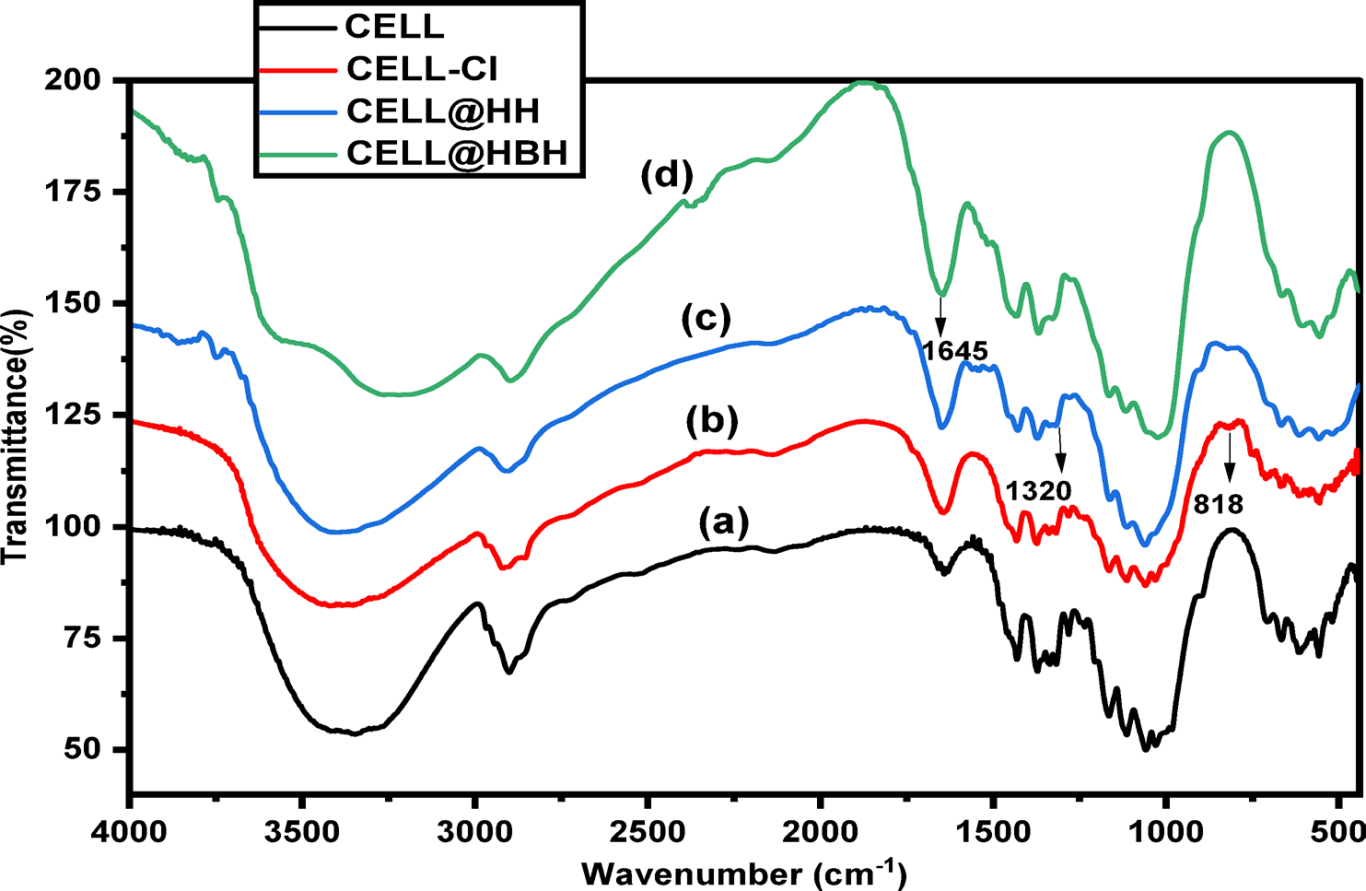
FTIR spectra of (a) CELL, (b) CELL-Cl, (c) CELL@HH, (d) CELL@HBH adsorbent.

FTIR spectra of native cellulose, its derivatives CELL-Cl, CELL@HH, and CELL@HBH are represented in Fig. 3.

The FTIR of CELL showed a number of distinctive peaks as the C–O stretching vibrations that appeared in the range of 1000–1200 cm^− 1^. Moreover, the peaks of C–H stretching vibrations that are present between 2700 and 3000 cm^− 1^^70^.

FTIR spectrum of CELL-Cl exhibits a new peak at about 818 cm^− 1^ which may be assigned to the C-Cl bond formed through the chlorination reaction^71^. Modification with hydrazide-hydrate results in appearance of new peak at 1320 cm^− 1^ which may be due to C-N stretching^72^, in addition to disappearing of peak of C-Cl at 818 cm^− 1^.

In CELL@HBH FT-IR spectrum, the broadness of 1645 cm^**− 1**^ peak increased which may be returned to C = N formation^73^ between the ligand’s aldehyde group and NH-NH_**2**_ group of CELL@HH. Moreover, the peak between 3005 and 3708 cm^**− 1**^ became broader due.

to increasing of OH groups resulted from interaction between ligand (DHB) and CELL@HH.

#### SEM

**Fig. 4:**
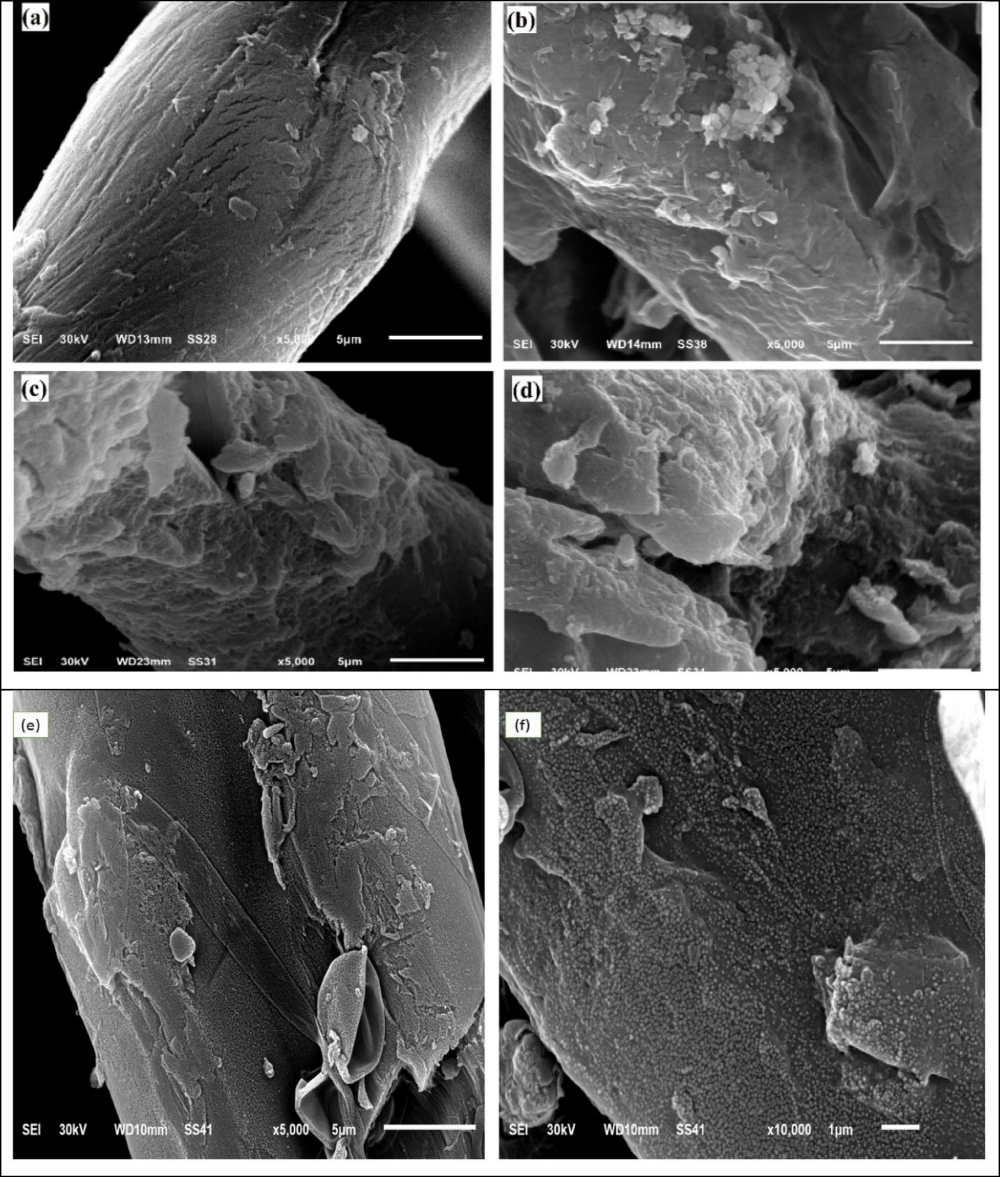
SEM images of: (**a**) CELL (**b**) CELL-Cl (**c**) CELL@HH, (**d**) CELL@HBH, (**e**) & (**f**) CELL@HBH@E122 at 5000x and 10000x magnification powers, respectively.

Scanning electron microscope had been used to visualize the morphological appearances of each of CELL (Fig. 4a), CELL-Cl (Fig. 4b), CELL@HH (Fig. 4c), and CELL@HBH (Fig. 4d). The images of SEM are displayed at magnifications of 5000X. The presence of both roughness and pores on the surface of the chlorinated cellulose may be due to chlorination action of the native cellulose with POCl_**3.**_ Increasing in the surface roughness without any cracking in cellulose structure itself after the treatment of chlorinated cellulose by hydrazine-hydrate and this may be returned to the insertion of hydrazine-hydrate instead of Cl atom. The surface then becomes rougher which may be attributed to the chemical reaction between the CELL-Cl and the CELL@HH to form CELL@HBH composite^74^. Figure 4e, f shows the SEM images of the CELL@HBH@E122 at two different magnification powers (5000x and 10000x). The observed full covering of the CELL@HBH surface with the E122 particles after the adsorption step confirms the adsorption process.

#### XRD

**Fig. 5:**
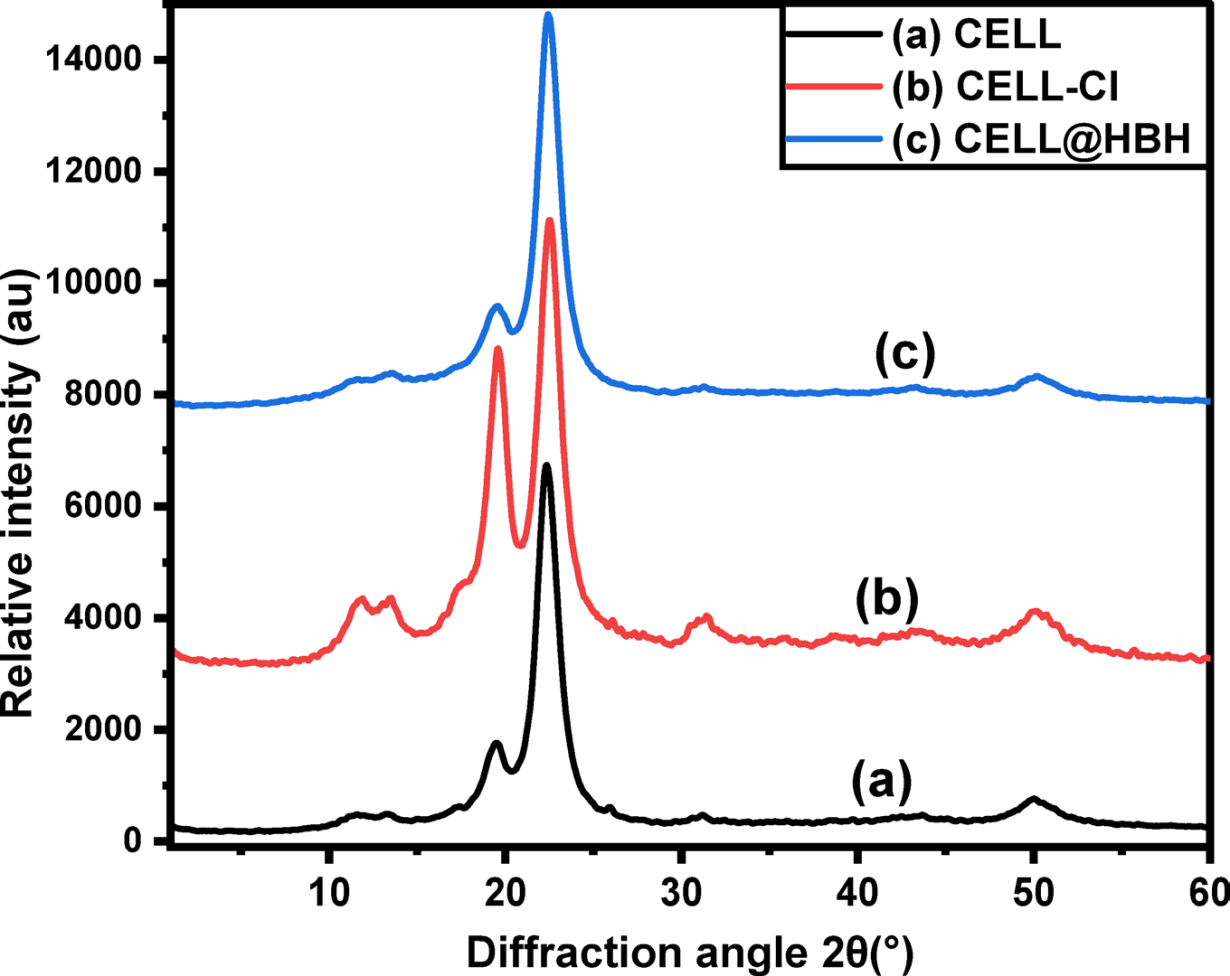
XRD patterns of: (a) CELL, (b) CELL-Cl and CELL@HBH sample.

Diffraction peaks were shown in XRD patterns in Fig. 5 at roughly 13.18º, 19.5º, 22.36º, and 31.18º. The primary peaks associated with crystallographic planes of (11̅0), (110), (200), and (004), respectively and these peaks were discovered to be related to cellulose^75^.

The locations of the studied peaks in CELL-Cl and CELL@HBH materials remain similar with a few minor adjustments. The crystallinity indices (CrI) of the samples were determined according to Eq. (19) using the Segal method^76^.

CrI% was found to be 92.7, 89.6, and 88.3% for native cellulose, CELL-Cl, CELL@HBH. A slight decrease in the crystallinity is observed which may be returned to that the main structure of cellulose doesn’t break down but only Cl atom replace OH group of cellulose in first step and then Cl atom replaced with hydrazine group in second step and finally ligand attached to N atom of hydrazine after removal of water molecule.

19 $$\:\text{C}\text{r}\text{I}\text{\%}=\:\frac{{\text{I}}_{200}\:-\:{\text{I}}_{\text{a}\text{m}}}{{\text{I}}_{200}}\:\text{x}\:100$$

Where, $\:{\text{I}}_{200}$ is the maximum intensity of the 002-lattice diffraction and $\:{\text{I}}_{\text{a}\text{m}}$is the intensity of diffraction at 2θ.

#### ^1^H NMR

The ^1^HNMR of CELL, CELL@HH and CELL@HBH were investigated by utilizing the DMSO/Trifluoro-acetic acid mixture. Figure represents the ^1^H NMR of CELL, CELL@HH and CELL@HBH. The ^1^HNMR of CELL present in (Fig.S2a) showed a peak at 2.03 ppm related to the proton that is present on C_2_ or C_3_^40^. ^1^HNMR of CELL@HH is represented in (Fig.S2b) that showed a new prominent peak, broad signal near 6 ppm that could be related to protons of NH and near 9 ppm may be related to NH_2_^77^. (Fig.S2c) represents the ^1^HNMR of CELL@HBH showed a new peak 6.5 to 7.5 ppm related to phenyl ring of induced ligand^78^.

#### TGA

**Fig. 6:**
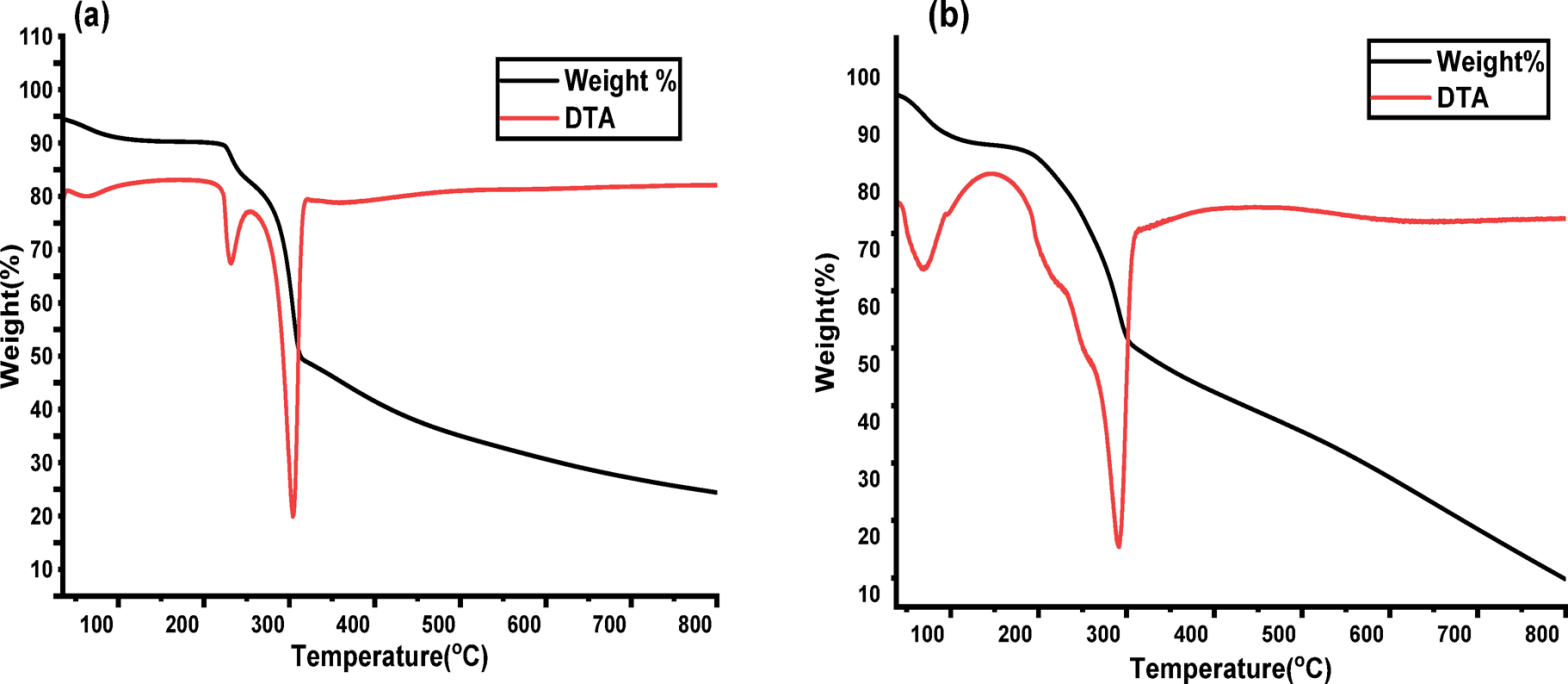
Thermal gravimetric analysis of: (**a**) CELL-Cl, (**b**) CELL@HBH.

The thermal degradation of CELL-Cl and CELL@HBH materials was investigated using thermogravimetry, as seen in Fig. 6a and b, respectively. The curve for CELL-Cl shows three mass losses corresponding to: (i) loss of water adsorbed on the surface in the 30–225 °C interval, (ii) mass losses attributed to loss of hydrochloric acid and condensation of hydroxyl groups present on carbons 2 and 3 in the 225 to 315 °C range, and (iii) decomposition of the organic framework above 315 to 800 °C. The CELL@HBH material also showed three mass.

losses, which were ascribed to water loss, pendant group degradation with condensation of DHB ligand hydroxyl groups and finally, breakdown of the organic support, respectively. At 800 °C, weight content decreased when the DHB ligand was loaded, passing from 29.409% for CELL-Cl to 14.77% for CELL@HBH material. proving that the DHB ligand accelerates the material’s degradation^79^.

### DFT calculations

#### Molecular geometry

**Fig. 7:**
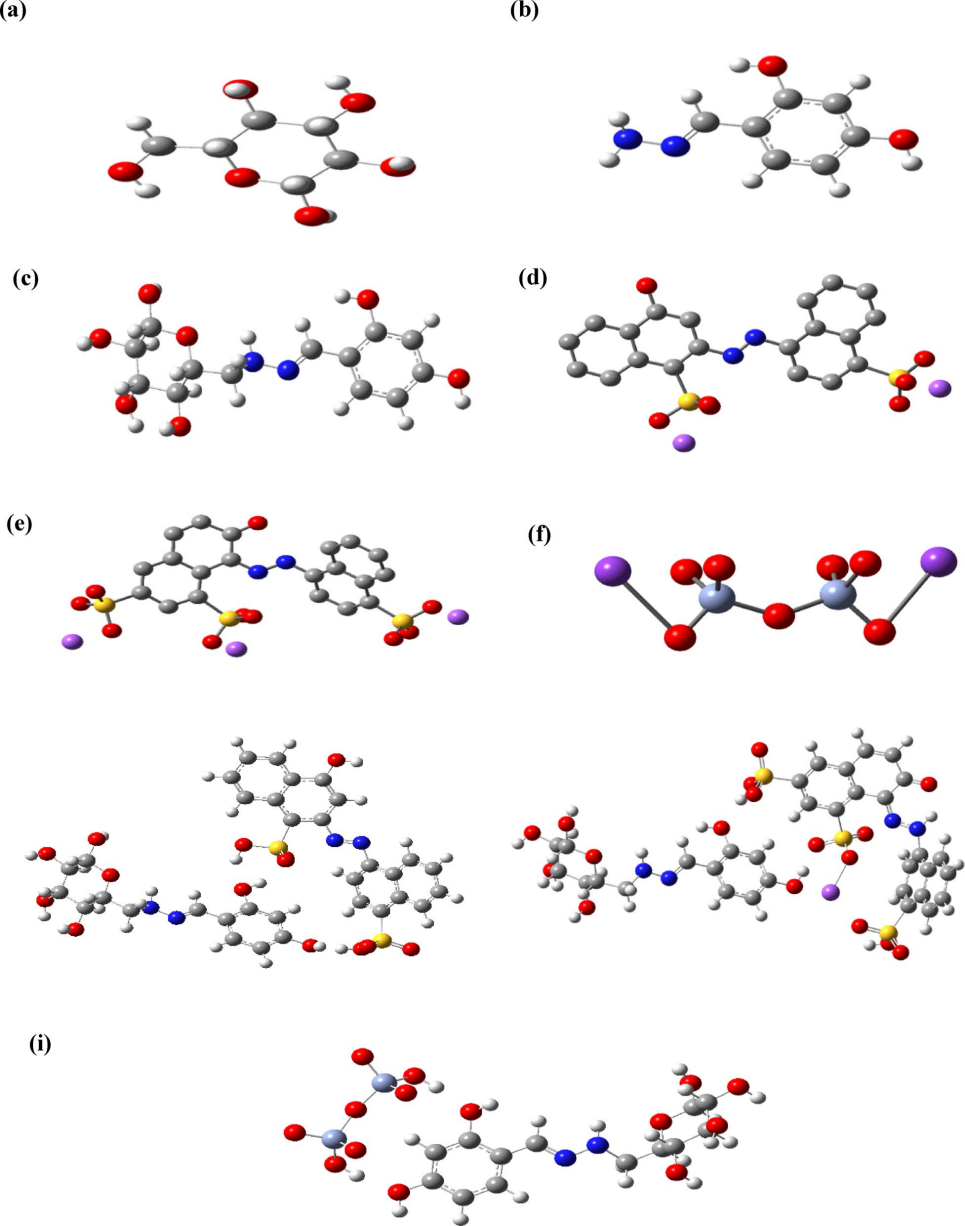
The optimized structures for (**a**) CELL, (**b**) HBH, (**c**) CELL@HBH, (**d**) E122, (**e**) E124, (**f**) Cr(VI), (**g**) CELL@HBH@E122, (**h**) CELL@HBH@E124 and (**i**) CELL@HBH@Cr(VI) based on the DFT/ B3LYP/6–31 g (d, p) methodology.

The optimized geometry of the CELL, HBH, CELL@HBH, E122, E124, Cr(VI), CELL@HBH@E122, CELL@HBH@E124, CELL@HBH@Cr(VI) are shown in Fig. 7.

#### Molecular orbital properties and global reactivity descriptors

**Fig. 8:**
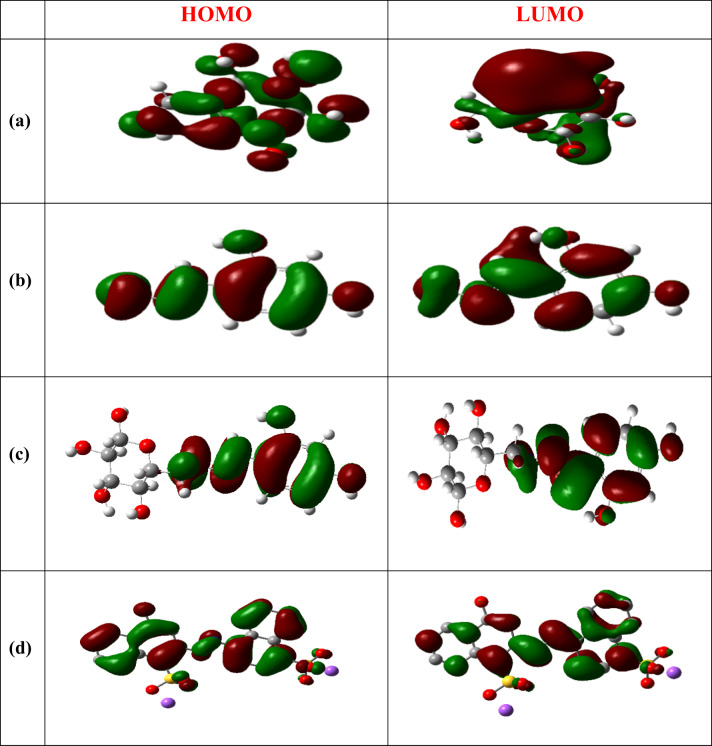

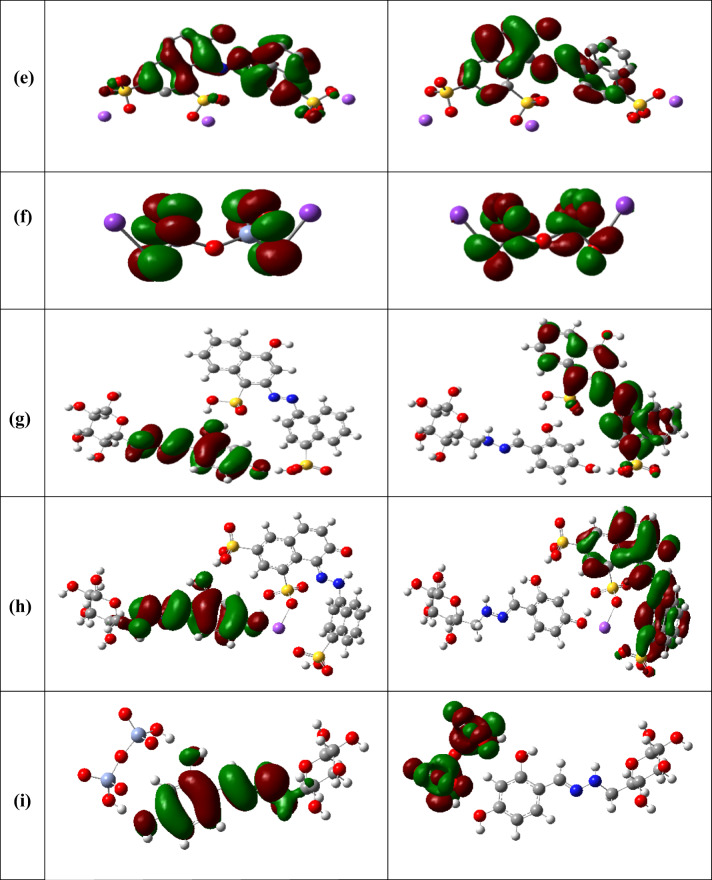
HOMO and LUMO structures for (**a**) CELL, (**b**) HBH, (**c**) CELL@HBH, (**d**) E122 (**e**) E124 (**f**) Cr(VI) (**g**) CELL@HBH@E122, (**h**) CELL@HBH@E124, (**i**) CELL@HBH@Cr(VI) based on the DFT/ B3LYP/6–31 g (d, p) methodology.

The Global Reactivity Parameters of a compound can be predicted from the HOMO-LUMO gap^80–82^. The HOMO is electron donor and LUMO the electron acceptor sites are shown in Fig. 8 for CELL, HBH, CELL-HBH, E122, E124, Cr(VI), CELL@HBH@E122, CELL@HBH@E124, CELL@HBH@Cr(VI).

Energy gap, ΔE_gap_, measures the reactivity; as the energy gap decreases the reactivity increases. The higher reactivity of CELL@HBH@E122, CELL@HBH@E124, CELL@HBH@Cr(VI) over CELL@HBH over HBH over CELL is cleared from the results of their ΔE_gap_ and the amount of electronic charge transferring as shown in Table S3.

Hard molecules ($\:\eta\:)$ exhibit a significant energy gap, while soft molecules ($\:\sigma\:$) possess a minor energy gap^83,84^. A soft molecule exhibits greater reactivity than a hard molecule due to its lower ΔE_(LUMO−HOMO)_. From the (σ) and (η) values shown in Table S3, CELL@HBH@E122, CELL@HBH@E124, and CELL@HBH@Cr(VI) are softer than CELL@HBH than HBH than CELL and this confirms the reactivity sequence of these molecules as mentioned above.

The $\:\chi\:$ is a measure of power of atom(s) to attract the electrons from the other molecules^85^. A high value of electronegativity (χ) for E122, E124, Cr(VI) suggest strong ability to attract electrons from CELL@HBH, which leads to greater interaction to form their products with CELL@HBH.

The ionization potential (I_P_) and the electron affinity (E_A_) can be expressed as negative values of E_HOMO_ and E_LUMO_, respectively. Greater ionization energy values signify increased stability and chemical inertness, whereas lower ionization energy values suggest greater reactivity of atoms and molecules^86^. The low ionization energy of CELL@HBH@E122, CELL@HBH@E124, and CELL@HBH@Cr(VI) than CELL@HBH than HBH than CELL indicates their higher reactivity in this order.

According to the definition electrophilicity index ( ω ) it measures the tendency of chemical species to acquire electrons. The greater electrophilicity values of CELL@HBH@Cr(VI), CELL@HBH@E122, CELL@HBH@E124 over CELL@HBH over HBH over CELL suggest strong ability of E122, E124, Cr(VI) to acquire electrons from CELL@HBH to form these products.

Finally, the dipole moment (µ) is a factor that can also provide information about interaction between molecules. The value (µ) of E122, E124, Cr(VI) product with CELL@HBH is higher than (µ) of E122, E124, Cr(VI) or CELL@HBH, this suggested the stronger interactions between them and CELL@HBH to form the compounds.

#### Molecular electrostatic potential (MEP)

A molecule’s electrostatic potential (MEP) map or surface illustrates the partial charge distribution that exists on it. Molecular electric potential surfaces, also known as electrostatic potential maps or electrostatic potential energy maps, offer a three-dimensional depiction of the charge distributions within molecules.

**Fig. 9:**
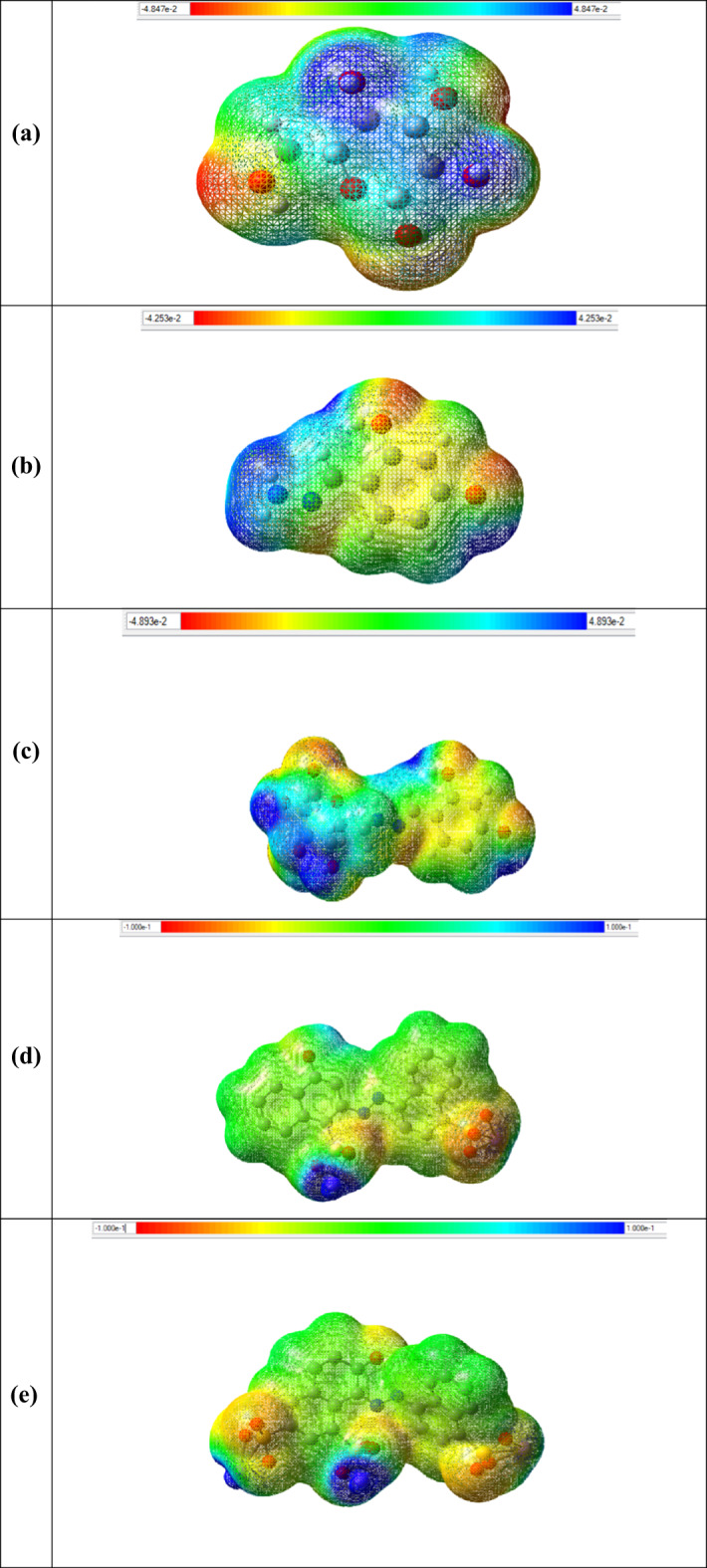

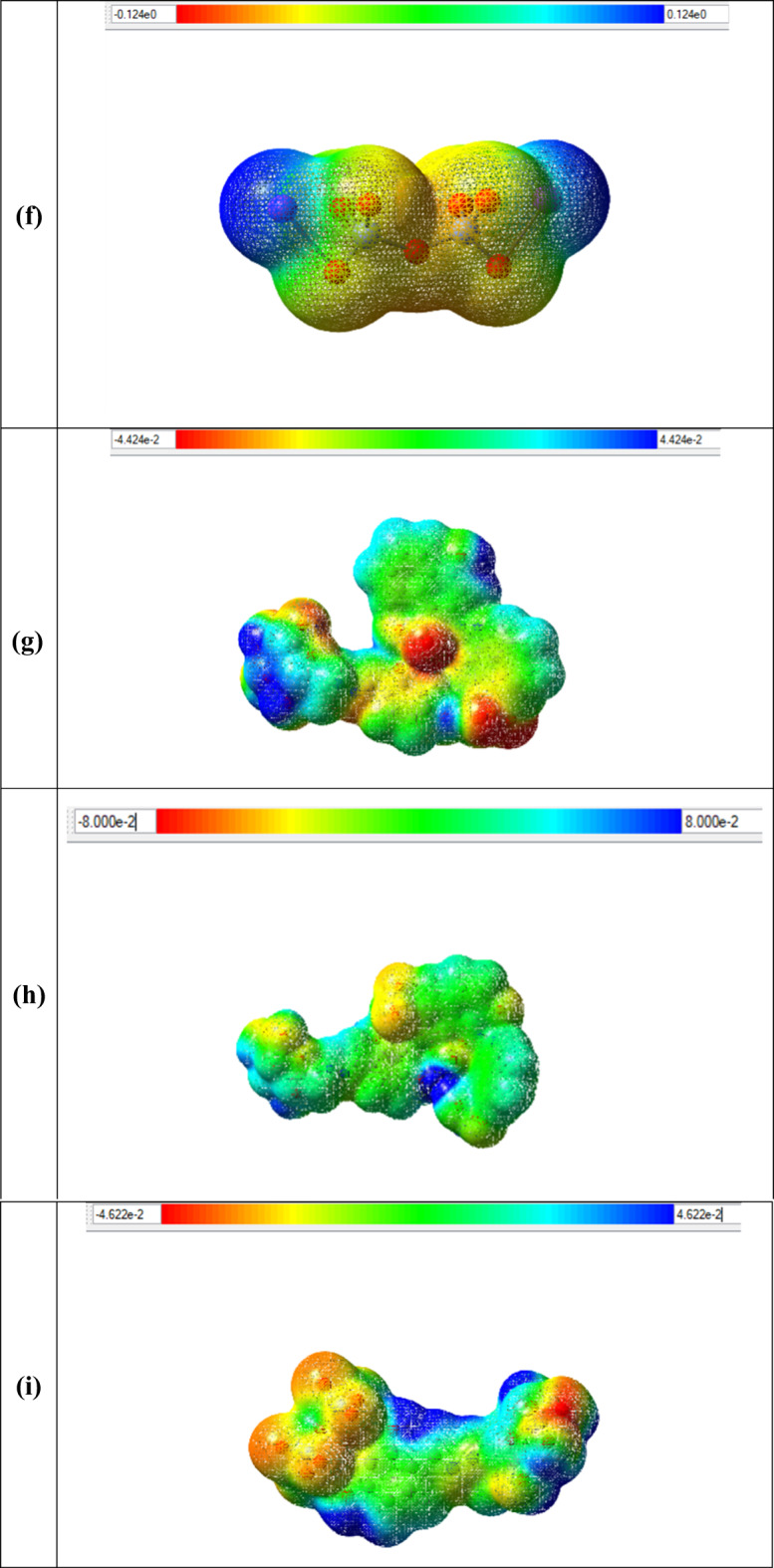
Electrostatic potential (ESP) surface maps visualization for (**a**) CELL, (**b**) HBH, (**c**) CELL@HBH, (**d**) E122, (**e**) E124, (**f**) Cr(VI), (**g**) CELL@HBH@E122, (**h**) CELL@HBH@E124, (**i**) CELL@HBH@Cr(VI) based on the DFT/ B3LYP/6–31 g (d, p) methodology based on the DFT/ B3LYP/6–31 g (d, p) methodology. Blue, green and red correspond to ESP varying from min to max level, the blue and red spheres correspond to ESP surface minima and maxima, respectively. ESP ranges are included in the legend at each figure panel.

These maps allow us to visualize variably charged regions of a molecule. Knowledge of the charge distributions are very useful to help determine molecular polarity and can be used to determine how molecules interact with one another. The color spectra are used to facilitate the interpretation of the electrostatic potential energy data, assigning red to represent the lowest value and blue for the highest, effectively illustrating the different intensities of these energy values. The calculated ESP maps of the Cellulose, CELL, HBH, CELL@HBH, E122, E124, and Cr(VI) and their product with CELL@HBH are shown in Fig. 9.

### Batch adsorption

#### Adsorption efficiency of raw cellulose and CELL@HBH adsorbent

**Fig. 10:**
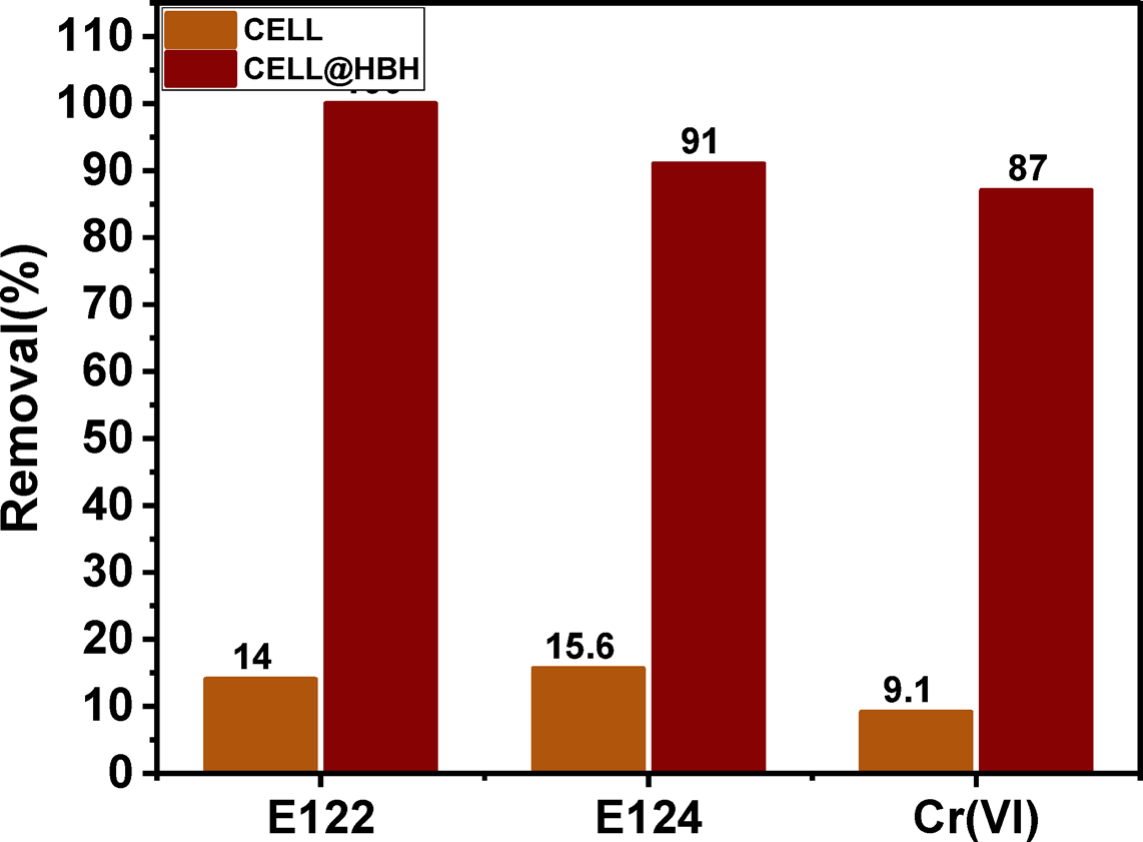
Comparison between the removal percentage (R%) of E122, E124, and Cr(VI) using native CELL and CELL@HBH adsorbent.

Preliminary experiments were performed to study the removal efficiency of raw cellulose and CELL@HBH adsorbent towards the removal of E122, E124, and Cr(VI) from aqueous solution at the optimum condition to demonstrate the benefits of the modification and the overall improvement in performance. The results are presented in Fig. 10. Under the same conditions for the investigated pollutants, the findings indicated that the effectiveness of the modification step with the ligand. The removal % utilizing raw cellulose (CELL) was 14%, 15.6%, and 9.1% for E122, E124, and Cr(VI), respectively. On the other hand, for CELL@HBH adsorbent, it was observed that the removal % increased to be 100%, 91%, and 87% for E122, E124, and Cr(VI), respectively. A finding that demonstrates the benefits of the modification and the overall improvement in performance.

#### Point of zero charge (pH_PZC_)

In order to understand the E122, E124, and Cr(VI) adsorption mechanism by the prepared composite, the point of zero charge of CELL@HBH material was studied. This investigation was obtained by measuring the pH at the point of zero charge (pH_**PZC**_). Commonly, the chelating agent will show greater affinities for anions at a pH value lower than the value of its pH_**PZC**_ and greater affinities for cations at a pH value higher than the value of its pH_**PZC**_. The pH_**PZC**_ value obtained for the CELL@HBH composite was approximately 5.92 as shown in Fig. S3. So, E122, E124, and Cr(VI) adsorption by the CELL@HBH was expected to be enhanced at a pH value lower than the pH_PZC_ value^87^.

#### Effect of pH

**Fig. 11:**
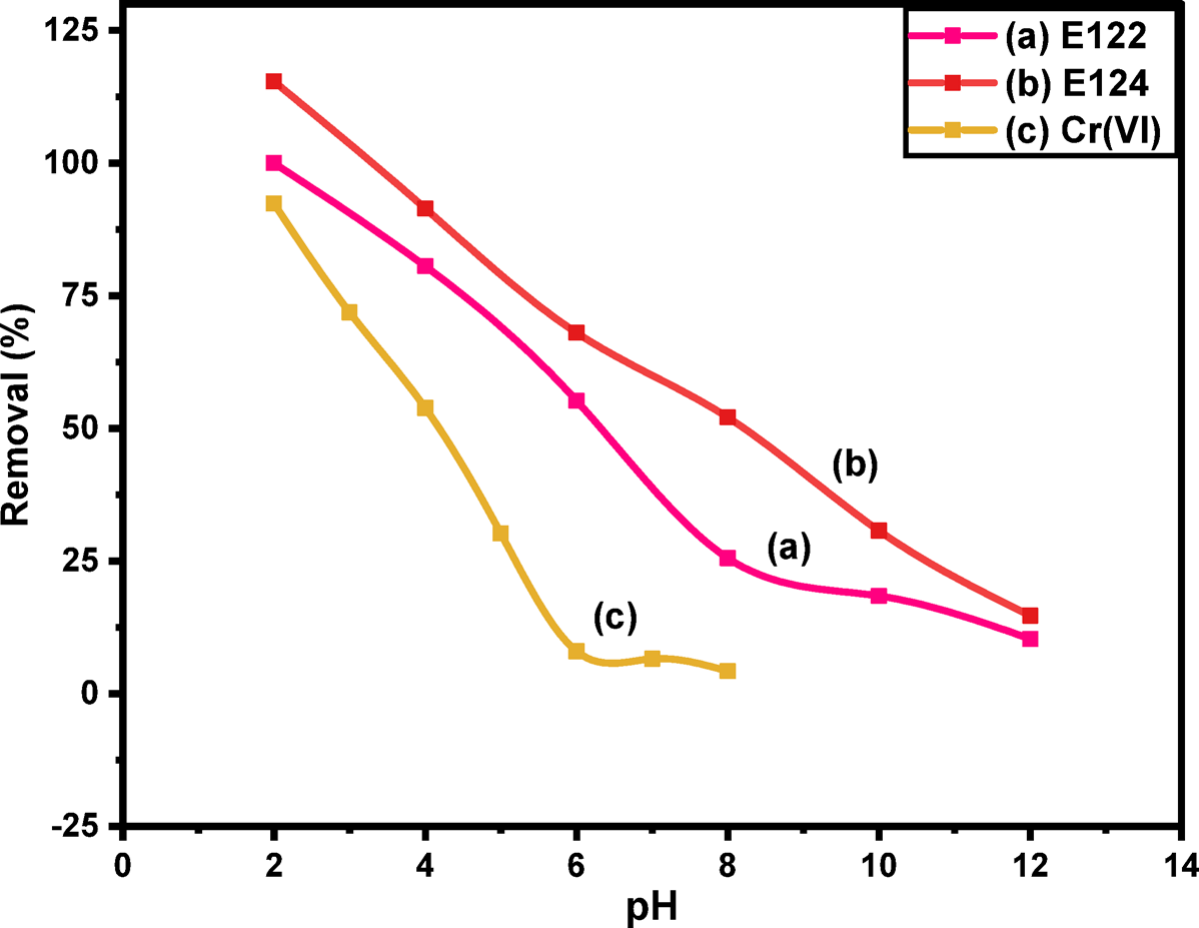
pH effect on adsorption efficiency of E122, E124, and Cr(VI) onto CELL@HBH under optimum conditions.) dose: 0.0075 g., conc.: 200 mg/L for E122, 150 mg/L for E124 and 100 mg/L for Cr(VI) in 20 mL volume).

By adding 0.0075 g of CELL@HBH to 20 mL of 200 mg/L and 150 mg/L for E122 and E124 aqueous solutions at 25 °C for 2 h, the pH parameter was investigated. The uptake of E122 and E124 was investigated in the pH range (2–12), as shown in Fig. 11. The maximum adsorption capacity for both E122 and E124 was found to be at pH 2 then drops from pH 4 to pH 12. pH parameter was also studied for Cr(VI) metal ion by adding 0.0075 g of CELL@HBH adsorbent to 20 mL of 100 mg/L Cr(VI) ion at 25° C for 5 h and it was discovered that pH 2 is the optimal pH value for the process of adsorption for Cr(VI) metal ion, followed by a fast drop from pH 4 to pH 6, then gradual decrease until it reaches the minimum value of adsorption capacity at pH 8.

The experiment results indicate that pH is a controlling factor in the adsorption mechanism as the uptake of the three pollutants increased whenever pH decreased. This can be explained by the fact that a decrease in solution pH leads to a rise in H^+^ ion concentration^88^.

While the anionic food colorant E122 and E124 and dichromate anion have a negative charge on their surfaces and pH produces a positive charge on the adsorbent surface, electrostatic attraction between the negative adsorbate and the positive adsorption sites occurred.

The pH_PZC_ of the CELL@HBH adsorbent, which is 5.92, is another finding that supports this conclusion. When pH of the solution is lower than this pH_PZC_, the adsorbent surface will have a positive charge, making it a surface that anions may adsorb onto.

#### Effect of dose

The adsorbent dosage is one of the important factors because it represents the capacity of the adsorbent for a given initial concentration of the adsorbate. It was investigated by adding various weights of CELL@HBH adsorbent in 20 mL aqueous solution of 200 mg/L E122, 150 mg/L E124 and 100 mg/L Cr(VI) at 25 °C for 2 h for both E122 and E124 and 5 h for Cr(VI) at pH 2 for all pollutants.

It was found that at low adsorbent dosage, the dispersion of CELL@HBH particles in the bulk solution is better; that is, all of the active sites on the adsorbent surface are completely uncovered which may accelerate the approachability of pollutants’ molecules to a large number of the adsorbent active sites. Therefore, the adsorption on the surface-active sites reaches a saturated point, performing a high removal capacity. However, at higher adsorbent dosage, the accessibility of adsorbent active sites with higher energy decreases and a larger portion of active sites with lower energy become occupied, leading to a decrease in adsorption process.

It was found that the ideal adsorbent dosage was around 0.0075 g for all of them as shown in Fig. S4 and the acquired results demonstrated that CELL@HBH is a highly effective adsorbent, even at extremely low dosages.

#### Effect of initial concentration and isotherm studies

Adsorption capabilities increased from 100 to 460 mg/g for E122 and from 266.7 to 339.4 mg/g for E124 as the initial concentration increased from 50 to 250 mg/L for E122 and from 100 to 200 mg/L for E124 and decreased from 266.667 to 198.986 mg/g for Cr(VI) as the initial concentration increased from 50 to 100 mg/L and then increased again from 198.896 to 300.065 mg/g between concentration of 100 to 250 mg/L. Furthermore, as the initial concentration grew from 250 to 300 mg/L for E122 and from 200 to 300 mg/L for E124 and 250 to 300 mg/L for Cr(VI), the adsorption abilities remained fairly stable. The results of the experiments varying the initial concentrations of the studied pollutants over the CELL@HBH samples are illustrated in Fig. S5.

When the initial concentrations of pollutants were increased, the adsorption capacities increased until they reached a maximum level at 200 mg/L, 150 mg/L, and 100 mg/L for E122, E124, and Cr(VI), respectively. The increase in the loading capacities of CELL@HBH adsorbent with increasing pollutants concentrations is due to the interaction between pollutants and the adsorbent which provides the vital driving force to defeat the resistances to the mass transfer of pollutants between the aqueous solution and the bone samples. Moreover, this phenomenon could be explained by the following reasons: at low pollutant CELL@HBH ratios, pollutant adsorption takes place on the high-energy sites, but upon increasing the pollutant CELL@HBH ratios ratio, the higher energy sites are saturated and adsorption commences on the lower energy sites, concluding in a decrease in adsorption efficiency^89–91^.

**Fig. 12:**
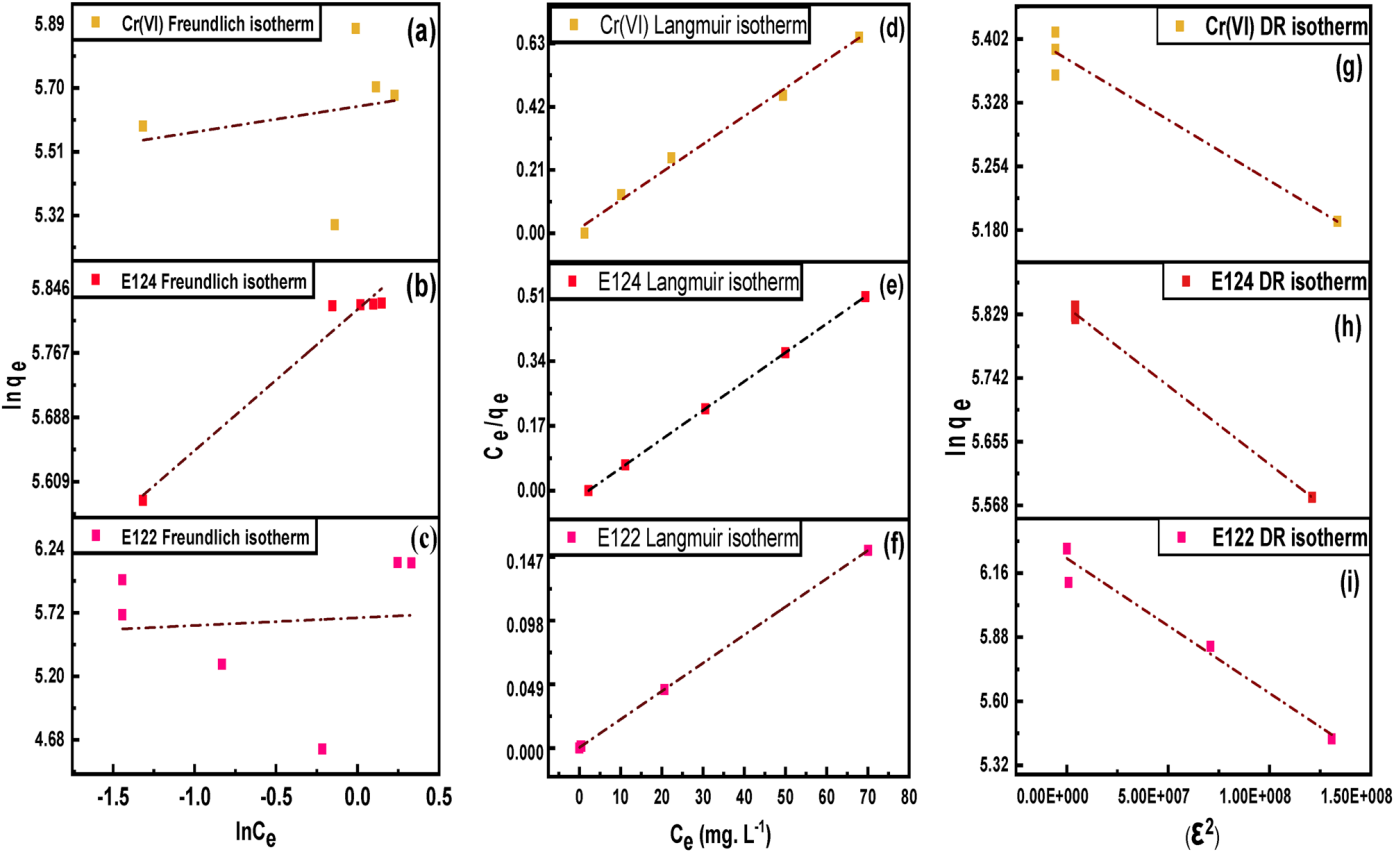
Freundlich isotherm model for (**a**) Cr(VI), (**b**) E124, (**c**) E122, Langmuir isotherm model for (**d**) Cr(VI), (**e**) E124, (**f**) E122, and D-R isotherm model for (**g**) Cr(VI), (**h**) E124, (**i**) E122.

According to the obtained results in Table S4, R^2^ values revealed a strong connection (0.99, close to 1) between the Langmuir model predicted and the experimental values, but the reverse was obtained by carrying out Freundlich model. The results showed that the adsorption of E122, E124, and Cr(VI) follows the Langmuir isotherm model due to the more subordinate error functions and more increased correlation coefficient (R^2^ ≥ 0.999). Hence, these results reflected that the experimental values of pollutants adsorption have a stronger correlation with the Langmuir isotherm model, as shown in Fig. 12.

The R_L_ values observed for all starting concentrations of Cr(VI), E122, and E124 are between 0 and 1. This means that the used adsorbent is suitable to be utilized for the removal of studied species.

The E_DR_ of adsorption from the Dubinin–Radushkevich model was calculated as 9.207, 8.935, and 9.532 kJ. mol^− 1^for E122, E124, and Cr(VI), respectively. These values suggested that the adsorption process of the three pollutants onto the CELL@HBH was carried out by a chemical mechanism in nature because the sorption energy lies within 8–16 kJ.mol^− 1^, showing that the adsorption process of E122, E124, and Cr(VI) on the CELL@HBH is chemisorption.

#### Effect of contact time and kinetic studies

As shown in Fig. S6 it was clear that the removal efficiency increased rapidly with the increase of contact time from 15 min to 120 min for both dyes, E122 (39.681 to 89.383%), E124 (49.122 to 84.711%), and from 120 to 300 min for Cr(VI) (62.567 to 73.262%). The efficiency remains constant when the shaking time is increased to more than 120 min for E122, E124, and more than 300 min for Cr(VI). This indicates that the removal efficiency reached equilibrium at 120 min for E122, E124, and 300 min for Cr(VI).

The initial rapid adsorption declines to a much lower rate approaching equilibrium and gradually levels off (120 min for E122, E124, and 300 min for Cr(VI)) towards the end of the experiment (a dynamic equilibrium is reached between CELL@HBH and the pollutants remaining in the liquid phase). In fact, the fast adsorption at the initial stage is probably due to the increased concentration gradient between the adsorbate in solution and in the adsorbent, as there must be an increased number of vacant active sites available at the beginning.

**Fig. 13:**
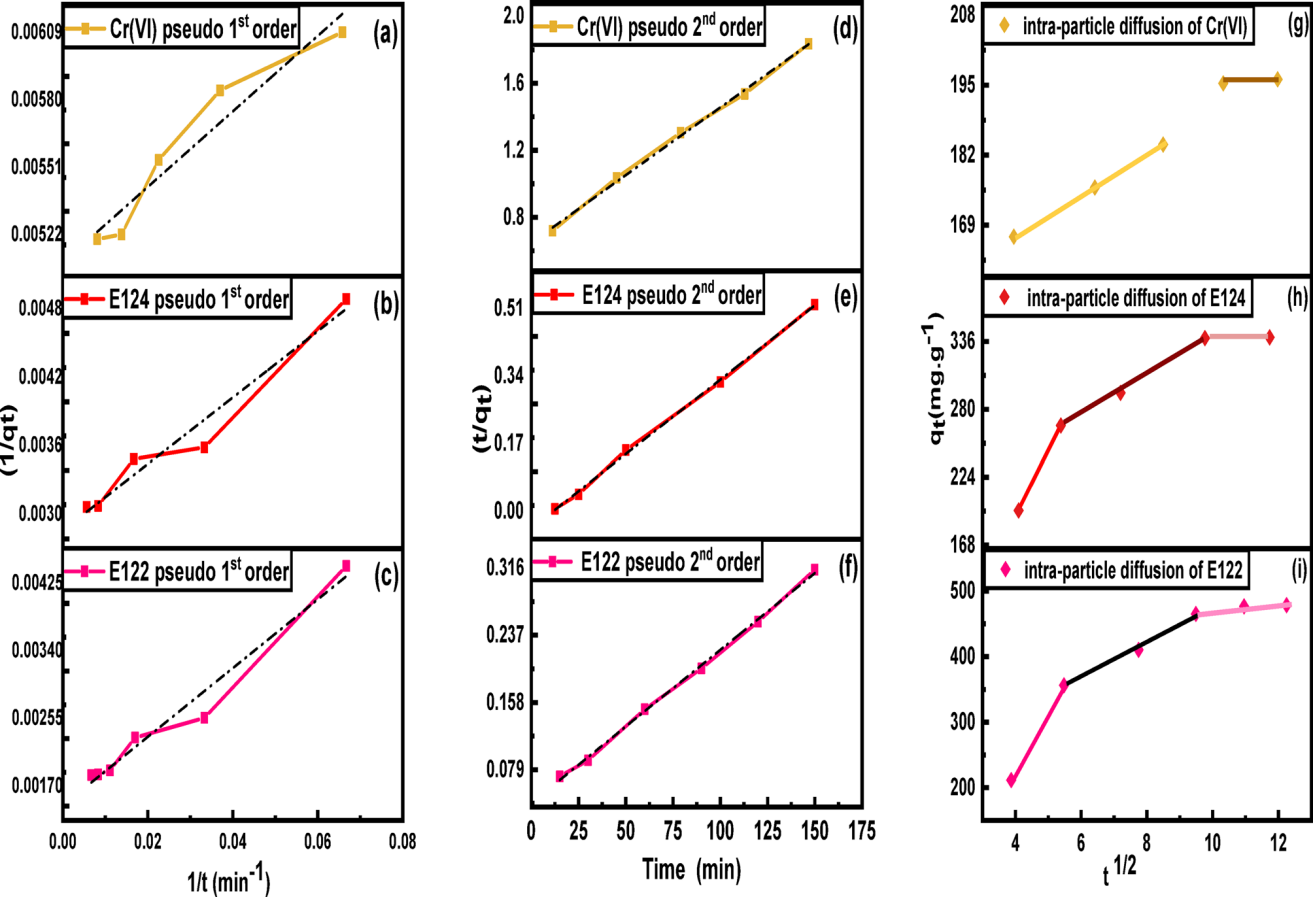
Pseudo-1st -order for (**a**) Cr(VI), (**b**) E122, (**c**) E124,  Pseudo-2nd -order for (**d**) Cr(VI), (**e**) E122, (**f**) E124, and IPD model for (**g**) Cr(VI), (**h**) E122, (**i**) E124 adsorption.

As in Fig. 13, three kinetic models including, pseudo-1st -order, pseudo-2nd -order, and intraparticle diffusion model (IPD) have been examined. Table 2 represents the estimated 1st -, 2nd -order, and IPD model parameters (K^1^, K^2^, K_diff_, q_e1_, and q_e2_) and correlation coefficient R^2^ values obtained from each investigated model nonlinear form.

The calculated adsorption capacities in the case of pseudo-2nd -order for E122, E124, and Cr(VI) were well fitted with the experimental values. Besides, R^2^ values of pseudo-1st -order were low: 0.96983 for E122, 0.96026 for E124, and 0.92890 for Cr(VI) in comparison with those of pseudo-2nd -order: 0.99827 for E122, 0.99889 for E124, and 0.99756 for Cr(VI). Therefore, pseudo-1st -order cannot be used for explaining the adsorption nature here. So, E122, E124, and Cr(VI) adsorption onto CELL@HBH adsorbent is well-fitted to pseudo-2nd -order.

The intraparticle diffusion plots for E122 and E124 indicated that the adsorption process occurred in three main stages. In stage 1, the slope was very sharp, as the dye molecules were quickly adsorbed onto the outer surface of the adsorbent as many active sites are present. This phase is called boundary layer diffusion, where the dye moves from the solution to the surface of the adsorbent. In stage 2, the line became straighter with a lower slope. This stage showed the dye molecules moving inside the pores of the adsorbent. This step is slower, as with time passing, the number of active sites on the CELL@HBH adsorbent decreases, and the diffusion of E122 and E124 into pores becomes more difficult. In stage 3, the slope became almost flat. This means the system was reaching equilibrium, and most of the active sites were full, and the adsorption slowed down a lot. But the intraparticle diffusion plot for Cr(VI) indicated that the adsorption process occurred in only two stages.

**Table 2:** Kinetic parameters for (E122, E124 and Cr(VI)) adsorption on CELL@HBH.

Adsorbates	Pseudo-1st-order		
	q_e_ (mg/g)	K_1_ (min^− 1^)	*R* ^2^
E122	584.795	25.26	0.96983
E124	325.773	9.47	0.96026
Cr(VI)	214.592	36.22	0.92890
	Pseudo-2nd-order		
E122	552.486	8.711 × 10^− 4^	0.99827
E124	321.543	3.536 × 10^− 4^	0.99889
Cr(VI)	219.298	1.08 × 10^− 4^	0.99756

Intraparticle diffusion model			
	K_diff_	*R* ^2^	
E122	29.705	0.85186	
E124	14.085	0.86023	
Cr(VI)	3.916	0.97166	

#### Effect of temperature and thermodynamic studies

It was noted that the adsorption capacity on CELL@HBH decreased with increasing temperature, as shown in Fig. S7 (a plot of ln K_C_ versus (1/T) temperature in Kelvin). Table 3 shows that the negative values of ΔG°_ads_ and ΔH°_ads_ indicate that adsorption of three pollutants on the surface of CELL@HBH is spontaneous and exothermic. The negative value of ΔS°_ads_ advertisements demonstrated an increase in order and a decrease in chaos.

**Table 3:** Thermodynamic parameters for the adsorption of E122, E124 and Cr(VI) on CELL@HBH adsorbent.

	T (K)	K_C_	ΔG°_ads_ (KJ/mol)	ΔH°_ads_ (KJ/mol)	ΔS°_ads_ (J/mol K)
E122	303	118.084	− 12.019	− 132.608	− 397.833
	308	55.249	− 10.274		
	313	23.939	− 8.397		
E124	303	33.295	− 8.829	− 88.378	− 262.697
	308	18.251	− 7.436		
	313	11.497	− 6.355		
Cr(VI)	303	24.143	− 8.021	− 89.625	− 269.024
	308	15.502	− 6.905		
	313	8.215	− 5.305		

#### Effect of ionic strength

A variety of species were utilized to investigate the ionic strength impact. This technique is critical because unfavorable ions exist in large amounts in industrial effluent. To investigate the influence of ionic strength, the following species were used: EDTA, CH_3_COONa, KCl, NaCl, and NaNO_**3**_ with concentration of 0.1 M and 1 M under optimal adsorption conditions by adding 0.0075 g of CELL@HBH to a 20 mL solution containing 200 mg/L of E122, 150 mg/L of E124, and 100 mg/L of Cr(VI) at 25 °C for 120 min for both E122 and E124 and 300 min for Cr(VI). As seen in (Fig. S8), the adsorption efficiency for the three pollutants increased as the concentrations of the studied electrolytes increased.

#### Desorption and regeneration studies

To test the reusability of CELL@HBH, the desorption procedure was performed under optimum parameters. Subsequently, we examined the result of applying adverse eluents like ethanol, EDTA(0.1 M), NaOH(0.5 M), HCl(0.5 M), Na_2_CO_3_(0.1 M) and CH_3_COONa(0.1 M) as presented in (Fig. S9) and it was observed that the effect of EDTA(0.1 M) achieved the best results for the desorption of E122 and E124 while NaOH (0.5 M) was the best eluent for Cr(VI). Hence, HCl, ethanol, Na_2_CO_3_, and CH_3_COONa are poorly affecting the desorption of the three investigated pollutants.

The CELL@HBH reuse was studied for five sorption–desorption cycles at the optimum conditions with sorption efficiency higher than 89% as shown in Fig. S10. It was predicted that CELL@HBH material could be a good sorbent for E122, E124, and Cr(VI) removal from aqueous solutions.

#### Removal of E122, E124, and Cr(VI) in multi-contaminant systems

**Fig. 14:**
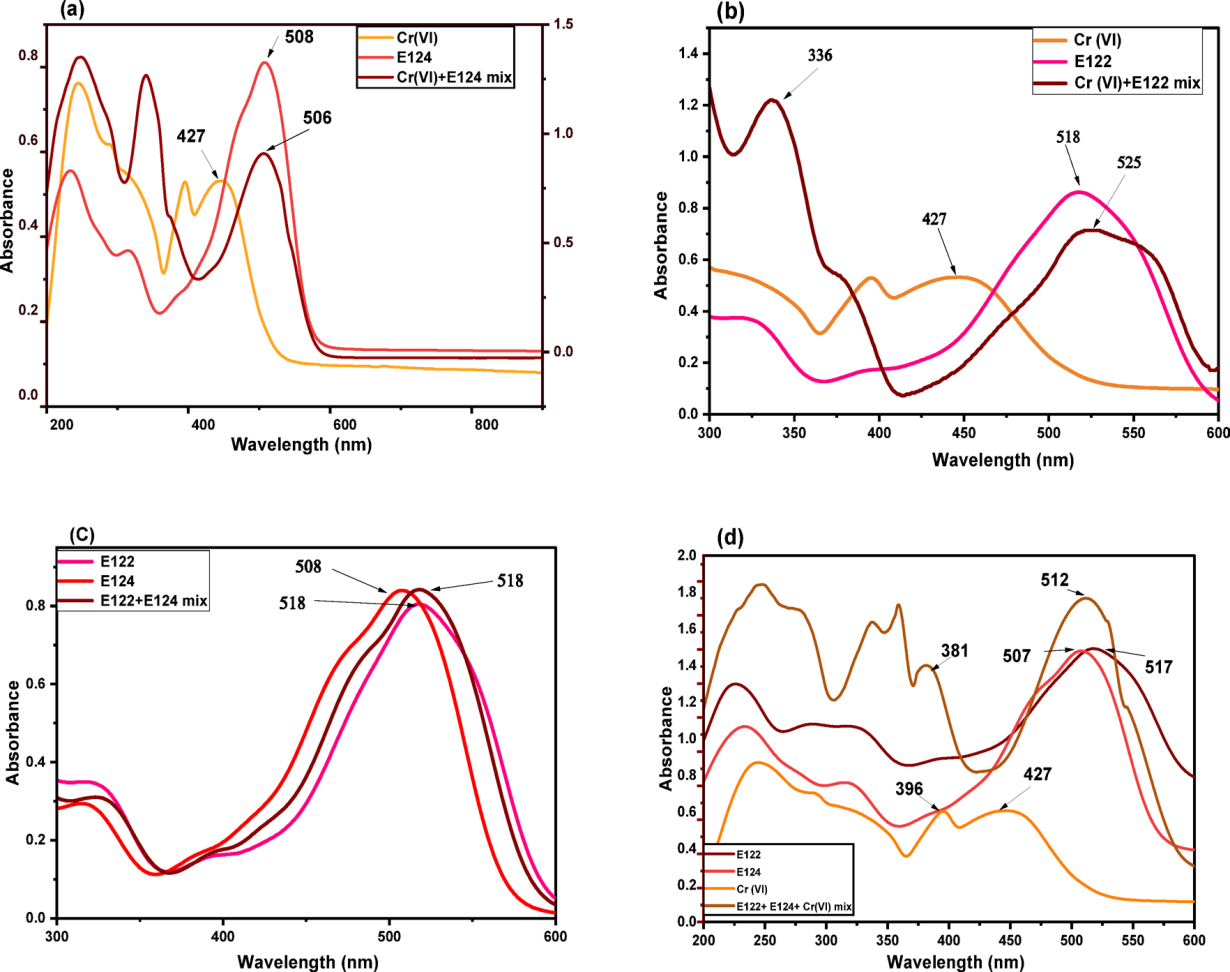
UV spectra of (**a**) Cr(VI) + E124 mix is compared with Cr(VI) and E124, (**b**) Cr(VI) + E122 mix is compared with Cr(VI) and E122, (**c**) E122 + E124 mix is compared with E122 and E124, and (**d**) E122-E124 + Cr(VI) mix is compared with E122, E124, and Cr(VI).

**Fig. 15:**
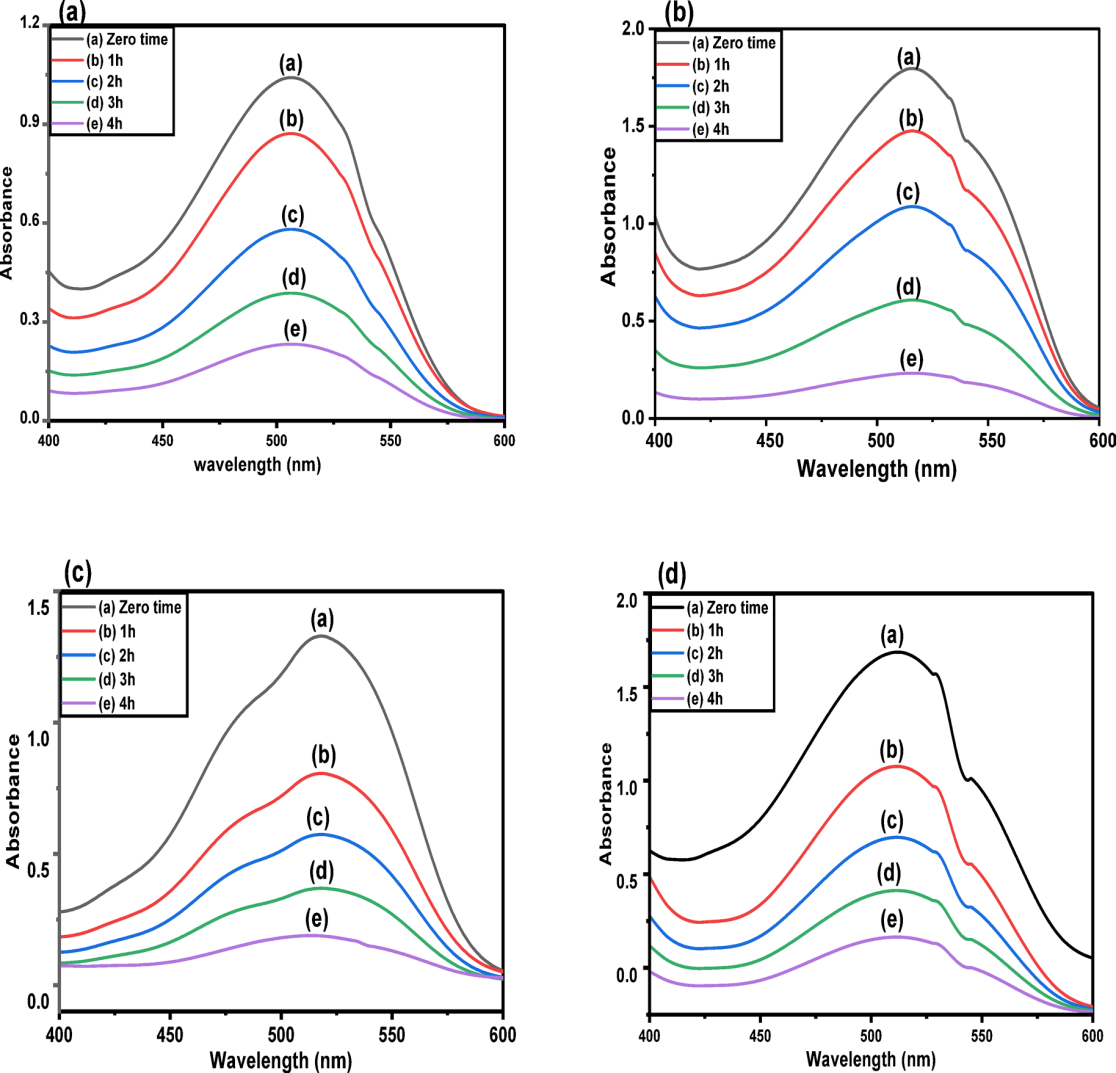
UV spectra of mixtures after adsorption by CELL@HBH at different periods of time: (**a**) Cr(VI) + E124 mix, (**b**) Cr(VI) + E122 mix, (**c**) E122 + E124 mix, and (**d**) E124 + E122 + Cr(VI) mix.

Figure 14a-d reflected that a new λ_max_ for each of the studied mixtures appeared that is not far away from the λ_max_ of the single pollutants (518 nm for E122, 508 nm for E124, and 427 nm for Cr(VI)), but in between, which emphasized the equivalent selectivity toward the removal of the three pollutants in the binary and multicomponent systems at equilibrium time as shown in Fig. 15a-d without forming an intermediate.

This behavior not only revealed the greater affinity for these three pollutants but also proved the absence of selectivity of the adsorbent toward adsorbing these pollutants in the bi- and multi-adsorbate systems. This might be attributed to the similarity in their anionic nature. In addition, the elimination capabilities of different combined contaminants under the same conditions were similar to q_e_ of individual E122, E124, and Cr(VI) (476.709, 338.789, and 190.072 mg/g). The results show that the prepared adsorbent was highly effective in removing a mix of anionic pollutants, proving its suitability for water treatment, particularly in systems with multiple pollutants. New overlapped peaks appeared after the formation of binary systems at 518 nm for [E122 + E124], 506 nm for [E124 + Cr(VI)], 525 nm for [E122 + Cr(VI)], and 512 nm for [E122 + E124 + Cr(VI)].

#### Application

##### In natural water samples

To evaluate the efficacy of modified cellulose for the adsorption of various contaminants, optimum experimental conditions were applied to natural samples. Standard solutions were utilized to generate calibration curves. Standard solutions (1.0 L) were handled using the optimal experimental conditions described above. Several water samples, including tap water from our lab and sea water from Ras El-bar City in Egypt, were utilized for analysis. Table S5 represents the results of the analysis. The recoveries of spiked samples containing known amounts of each contaminant were studied. The recoverable values varied between 96.00 and 118.101% with RSD < 1.50. These results indicate that modified cellulose can reliably recognize E122, E124, and Cr(VI) in natural water.

##### In colored soft drinks and food industries 

To evaluate modified cellulose performance for anionic dye adsorption, several samples containing E122 and E124 were exposed to the best experimental conditions for both. The calibration curves were created using standard solutions. The dietary samples consist of soft drinks and jelly. Fig.S11 reveals that over 97.5% of the E122 and E124 removal from the tested samples was successful. These data show that CELL@HBH adsorbent may be used to remove E122 and E124 from a variety of materials.

#### Performance of CELL@HBH adsorbent

The E122, E124, and Cr(VI) anionic species can be effectively separated and removed from a variety of water samples using the CELL@HBH adsorbent. A comparison of the CELL@HBH adsorbent performance with those of the other cited adsorbents is shown in Table 4. When comparing various adsorbents for the remediation of E122, E124, and Cr(VI), it was found that the sorption capacity, type of adsorbent, adsorbent dose, initial concentration, and equilibrium time at which the sorption is performed should be considered. Finally, Table 4 shows that, in comparison to the other adsorbents mentioned, the CELL@HBH has high capacities and efficiencies for the E122, E124, and Cr(VI).

**Table 4:** Comparison of adsorption capacity of E110, E122, E124, and Cr(VI) onto CELL@HBH with previously reported studies.

Adsorbate	Adsorbent	Adsorbent dose	Initial concentration (mg/L)	Equilibrium time (min)	Adsorption capacity (mg/g)	References
E122	CELL@HBH	0.0075 g	200	120	476.709	Present study
	Zn-Al- LDH (layered double hydroxides) clay	0.035 g	20	40	21.46	^92^
	Natural-diatomite (ND)	1 g	50	30	12	^93^
	Doped (Ag) on zinc oxide nanoparticles (ZnO NPs)	0.08 g	5	80	84.7	^94^
E124	CELL@HBH	0.01 g	150	120	338.789	Present study
	Fe_3_O_4_@SiO_2_-CMK-8, MNCs	0.01 g	50	20	78.74	^95^
	NH2-MMNC	0.08 g	50	30	58.8	^96^
Cr(VI)	CELL@HBH	0.0075 g	100	300	190.072	Present study
	activated carbon derived from paper mill sludge by ZnCl_2_	3.5 g/L	100	180	23.18	^97^
	MCC/PANI-69 wt% nanocomposite	4 g/L	100	30	35.97	^98^
	Pine sawdust cellulose fibres	2 g	100	90	9.638	^99^

#### Plausible mechanism of adsorption onto CELL@HBH

**Fig. 16:**
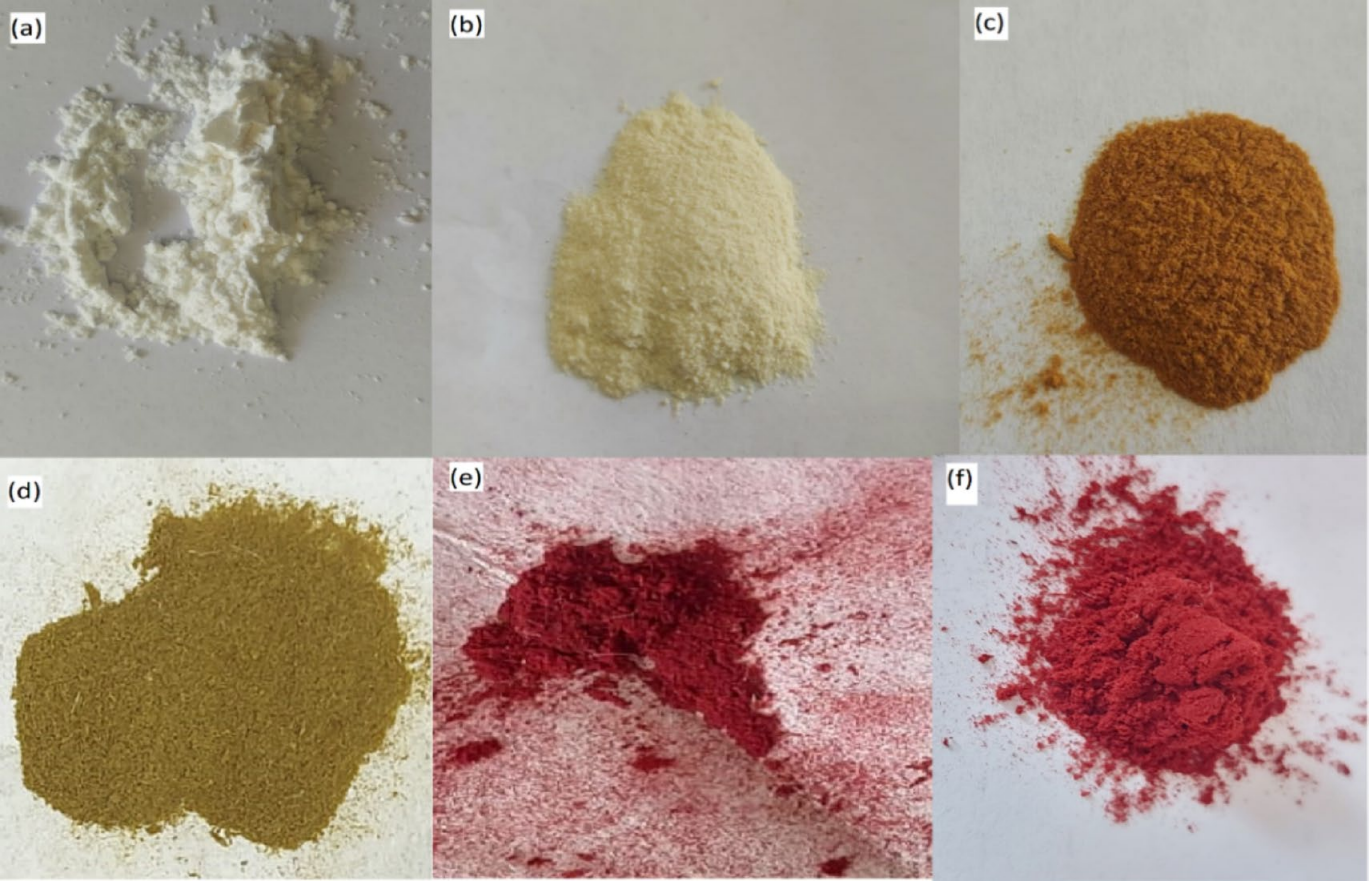
Digital photographs of (**a**) CELL (**b**) CELL@HH (**c**) CELL@HBH (**d**) CELL@HBH@Cr(VI) (**e**) CELL@HBH@E122 (**f**) CELL@HBH@E124.

The plausible mechanism can be discussed based on the optical images of CELL@HBH before and after adsorption of pollutants; the digital photographs of CELL, CELL-Cl, CELL@HH, and CELL@HBH adsorbent are shown in Fig. 16, respectively. An obvious color change after each step, it converted from the white color of the CELL (Fig. 16a) to the pale-yellow color after modification with hydrazide hydrate (Fig. 16b) and to brown after modification with 2,4 Di-Hydroxy Benzaldehyde (DHB) ligand as in (Fig. 16c). The color of the CELL@HBH changed into greenish yellow after the adsorption of Cr(VI) as in (Fig. 16d). And to dark red and red color after the adsorption of E122 and E124, respectively (Fig. 16e), (Fig. 16f).

The prediction of the adsorption mechanism of the anionic species was based on the functional group present on the surface of CELL@HBH, as illustrated in Fig.S12a, b, and c. This functional group is the hydroxyl group (-OH). In Fig.S12a, it can be seen that Cr(VI) is adsorbed by CELL@HBH in an acidic environment through the electrostatic attraction between the negatively charged oxygen in Cr(VI) and the positively charged groups (-OH^2+^) present in the CELL@HBH adsorbent. Figure S12 b, c shows that the adsorption of anionic food dyes (E122 and E124) wasn’t attributed only to electrostatic attraction reactions but also to the n-π interactions between electron-donating groups, such as oxygen groups in the adsorbent and aromatic rings in both dyes^100,101^. The FT-IR spectra after loading of E122, E124, and Cr(VI) are shown in Fig.S12d. Decreasing in the broadness of the hydroxyl group can be observed in the IR spectra of CELL@HBH@Cr(VI), CELL@HBH@E122, and CELL@HBH@E124 indicating the reaction between + ve charge on (-OH_2_^+^) of the CELL@HBH adsorbent and -ve charge on E122, E124 and Cr(VI) through electrostatic attraction in addition to slight shift of this OH group band from 3216 cm^**− 1**^ to 3362 cm^− 1^ for both CELL@HBH@E122 and CELL@HBH@E124 and to 3421 cm^− 1^ for CELL@HBH@Cr(VI). In the IR spectra of CELL@HBH@E122 and CELL@HBH@E124, new peaks appeared at 1282 cm^− 1^, attributed to (S = O) stretching of the (SO_**3**_) group. Another new peak can be observed at 1505 cm^− 1^ refers to the C = C of the alkene of aromatic structure^102^. In the IR spectra of CELL@HBH@Cr(VI), the peak of Cr(VI) was supposed to be found in the range of 500–600 cm^− 1^, but it wasn’t visible individually due to the presence of overlapping bands in this region (fingerprint region of cellulose material).

### Future prospectives 

The current investigation demonstrated the preparation of cellulose-based adsorbents for the removal of both organic (E122 and E124) and inorganic (Cr(VI)) species in single and multiple systems. Moreover, several aspects should be taken into consideration for more advanced research:

1. The adsorption of pollutants in multiple systems containing both organic and inorganic contaminants is a vital issue, as this simulates the actual case investigation of real water treatment.
2. Error functions calculation for the isotherm models should be obtained for evaluating the adsorption process with well-fitted models.
3. The application to real water samples should be taken into consideration.
4. Throwing light on the characterization of the adsorbent after the adsorption process, for further confirmation of the adsorption process.
5. Giving more attention to the adsorbent reusability.

### Advantages and limitations of the current investigation

The current investigation demonstrates various advantges, including (i) the removal of organic and inorganic pollutants effectively in single and multiple systems, (ii) the characterization of the CELL@HBH before and after the adsorption step is obtained, (iii) the high adsorption capacity for the investigated pollutants, (iv) the application of the prepared material on spiked real water samples, and (v) the cost affordability.

Any water treatment process may be accompanied by various limitations, like selectivity, sensitivity, adsorption efficiency, adsorbent stability, regeneration, cost, and environmental challenges^103^. The limitations of the current study are (i) this investigation lacks a long-term biodegradability study, (ii) it is limited to batch adsorption, lacks a column investigation, and (iii) the regeneration investigation is limited to 5 cycles; an extended regeneration investigation is needed.

## Conclusion

Here, the CELL@HBH adsorbent is a highly effective material for the adsorption of E122, E124, and Cr(VI)  from aqueous solutions. The CELL@HBH adsorbent achieved high adsorption capacity at optimal conditions of 476.709 mg/g, 338.789 mg/g, and 190.072 mg/g for E122, E124, and Cr(VI) , respectively. It was discovered that the adsorption of the investigated pollutants depended on oscillating time, initial concentration, CELL@HBH dose, initial solution pH, and temperature. The results showed that the adsorption of E122, E124, and Cr(VI) follows pseudo-2nd-order kinetic and Langmuir isotherm models due to the more subordinate error functions and more increased correlation coefficient (R^2^ ≥ 0.999) indicating that the adsorption of E122, E124, and Cr(VI) onto CELL@HBH adsprbent is chemisorption in a monolayer patteren. Thermodynamic investigations indicate that the adsorption mechanism is exothermic and spontaneous from -ve ΔG° and ΔH° values. In contrast to earlier research that concentrated on single pollutant systems, this investigation is unique in that it applies CELL@HBH for the simultaneous removal of several pollutants (E122, E124, and Cr(VI)) in single and mixed systems. This makes the substance a flexible option for real-world wastewater treatment situations requiring convoluted pollutant blends. The CELL@HBH adsorbent has good recycling performance. Under five regeneration and adsorption cycles, it still has removal effect greater than 85% of E122, E124, and Cr(VI), which indicates its high structural stability. The adsorption mechanism of E122, E124, and Cr(VI) onto CELL@HBH is elucidated. Ultimately, this study demonstrates that the fast-responsive CELL@HBH can be effectively utilized to eliminate E122, E124, and Cr(VI) from a wide range of real water sources. Collectively, the results indicate that the as-prepared CELL@HBH is promising for anionic pollutant adsorption and our mechanistic results are of guiding significance in environmental cleanup.This work contributes significantly to understanding how experimental conditions influence the mechanism of E122, E124, and Cr(VI) adsorption by CELL@HBH adsorbent, offering valuable and new insights for future applications and optimizations in the treatment of effluent-containing anionic species. Eventually, the eco-friendly CELL@HBH adsorbent can be a viable option for eliminating anionic pollutants from aquatic systems and industrial samples.

## Supplementary Information

Below is the link to the electronic supplementary material.

Supplementary Material 1

## Data Availability

Data is provided within the manuscript or supplementary information files.
