# Supplementary material for: Novel dihydroxybenzohydrazide grafted deoxycellulose for efficient removal of anionic food colorants and hexavalent chromium from wastewater
Source: Sci Rep. 2025 Aug 13;15:29751. doi: 10.1038/s41598-025-14609-5 (PMC12350884; doi:10.1038/s41598-025-14609-5)
Supplement: Supplementary file 1 — Supplementary Material 1 [file 41598_2025_14609_MOESM1_ESM.docx]

**Novel dihydroxybenzohydrazide grafted deoxycellulose for efficient removal of anionic** **food colorants and hexavalent chromium from wastewater**

**Magda A Akl^1^*, Azza AH Fahim^1^ and Elsayed RH El-Gharkawy^1^**

**^1^**Department of Chemistry, Faculty of Science, Mansoura University, Mansoura 31556, Egypt

* To whom correspondence should be addressed: Prof Magda Akl.

email magdaakl@yahoo.com

|  |  |
| --- | --- |

**Figure S1.** BET of: (a) CELL-Cl, and (b) CELL@HBH.

| 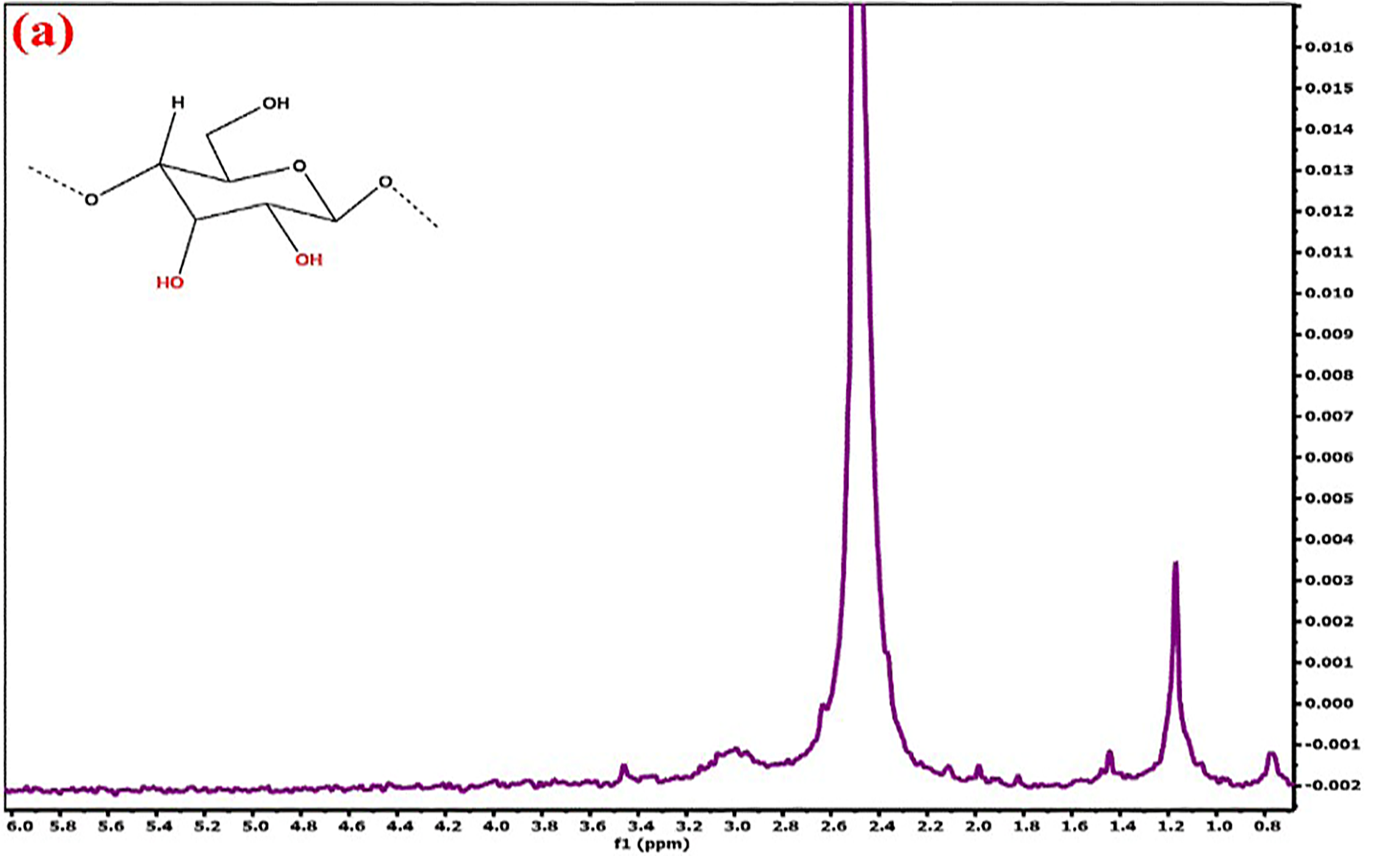  DMSO |
| --- |
| 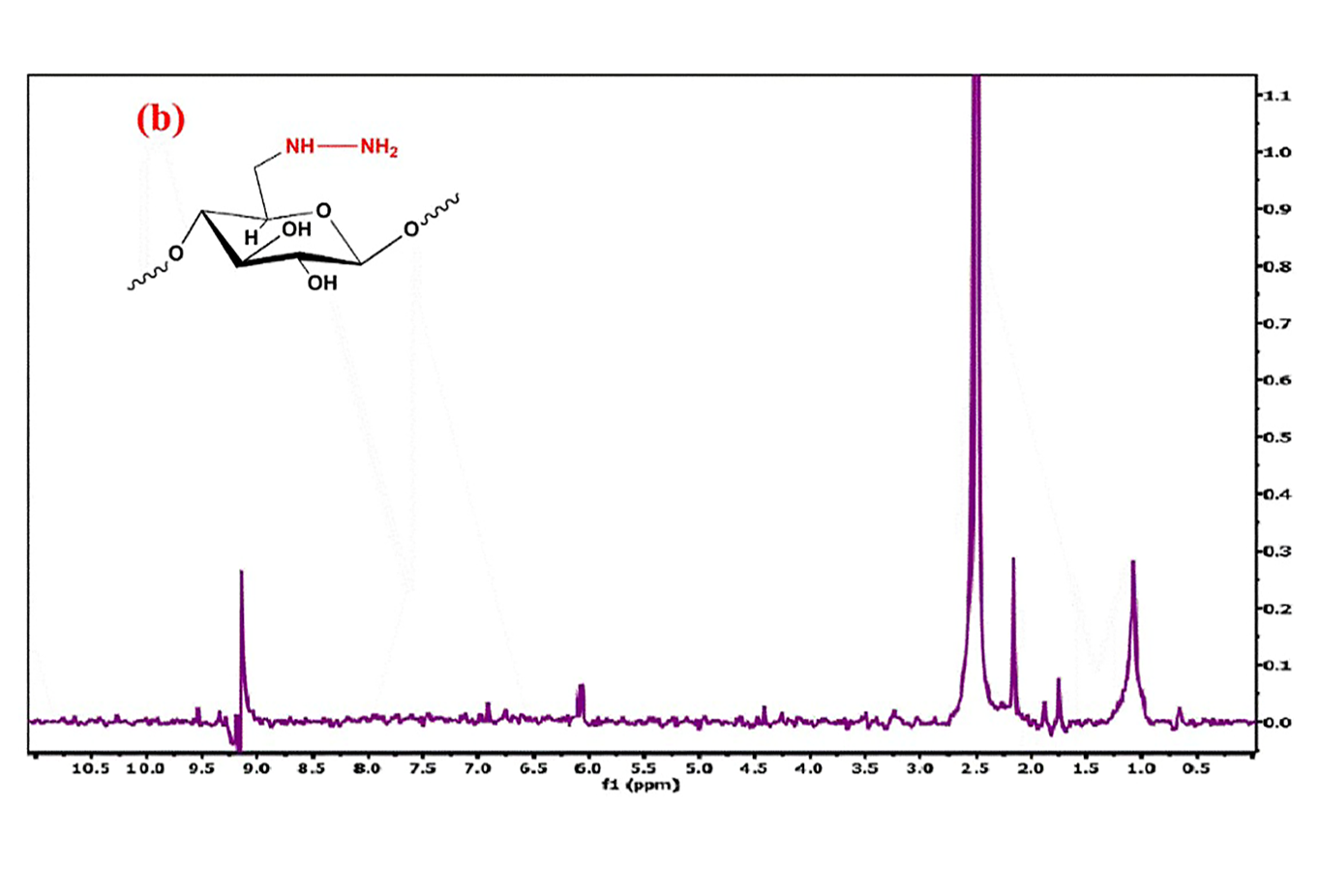  DMSO |
| 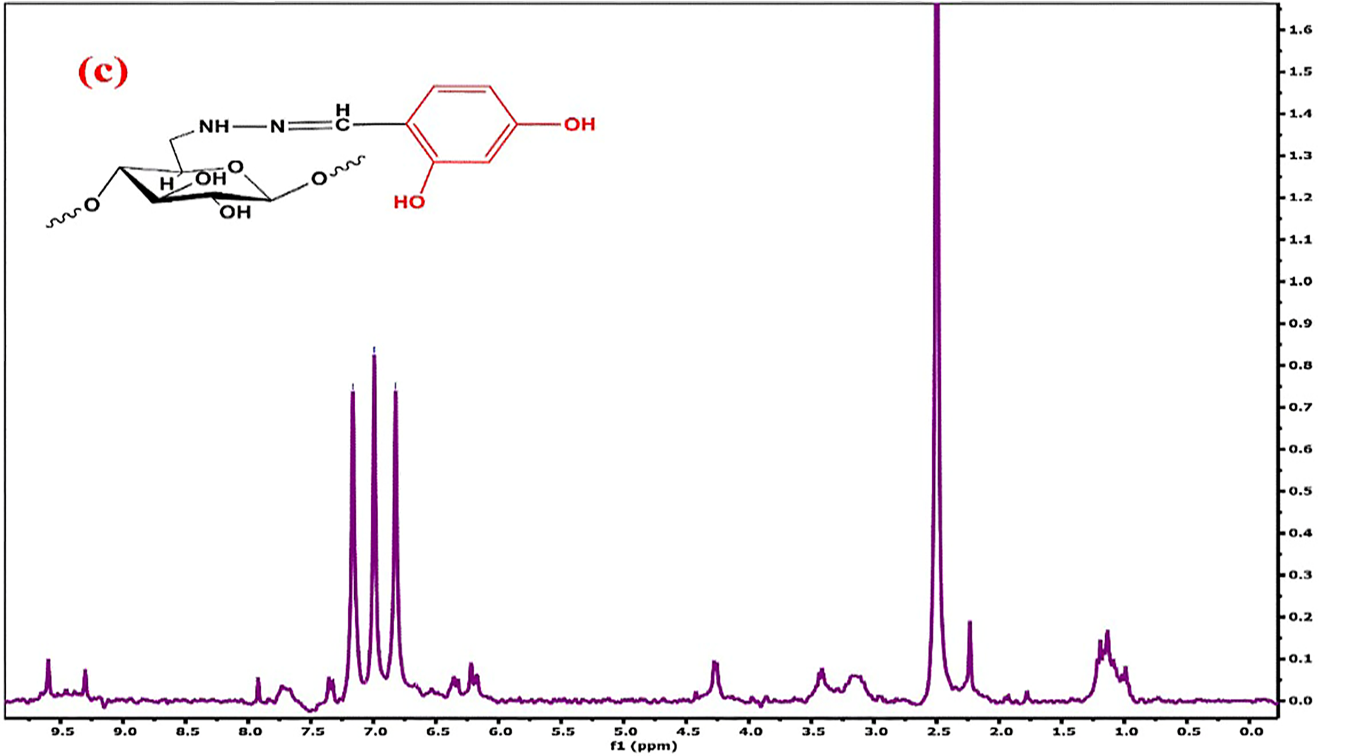  DMSO |

**Figure S2**. ^1^ H NMR of (a) CELL, (b) CELL@HH and (c) CELL@HBH

**Figure S3.** Point of zero charge of CELL@HBH

|  |  | |
| --- | --- | --- |
|  | |  |

**Figure S4**. Effect of dose of: (a) Cr(VI) (100 ppm), (b) E124 (150 ppm), and (c) E122 (200 ppm) adsorption on CELL@HBH in 20ml at room temperature.

|  |  |
| --- | --- |
|  | |

**Figure S5.** Effect of initial concentration of: (a) Cr(VI), (b) E124, and (c) E122 on adsorption efficiency

**Figure S6.** Effect of time on (a) E122, (b) E124, and (c) Cr(VI) adsorption efficiency on CELL@HBH adsorbent under optimum conditions.

|  |
| --- |

**Figure S7.** Plot of ln K_C_ versus (1/T) absolute temperature for the adsorption of (a) E122, (b) E124, (c) Cr(VI) on the surface of CELL@HBH.

**Figure S8.** Effect of ionic strength on E122, E124, and Cr(VI) adsorption on CELL@HBH.

**Figure S9.** Desorption of E122, E124, and Cr(VI) from CELL@HBH adsorbent by different eluents.

**Figure S10.** Repeated 5 cycles of E122, E124, and Cr(VI) adsorption–desorption using EDTA(0.1M) for E122 and E124 and NaOH (0.5M) for Cr(VI) at room temperature.

**Figure S11.** Recovery (%) of 200 ppm E122 from (A) strawberry tasted Gum, (B) pomegranate flavor soft drink and 150 ppm E124 from (C) jelly.

|   **(a)** |
| --- |
|   **(b)** |
|   **(c)** |
|   **(d)** |

**Figure S12.** schematic illustration of **(a)** Cr(VI), **(b)** E122, **(c)** E124 adsorption on the CELL@HBH adsorbent. **(d**) FT-IR spectra of **(d1)** CELL@HBH **(d2)** CELL@HBH@E122 **(d3)** CELL@HBH@E124 **(d4)** CELL@HBH@Cr(VI)**.**

**Table S1.** The Global Reactivity Descriptors (GRD)

| **GRD** | **Equation** |
| --- | --- |
| Ionization potential | I_P_ = - E_HOMO_ |
| Electron affinity | E_A_ = - ELUMO |
| The energy gap | ΔE_gap_ = E_LUMO_ - E_HOMO_ |
| The hardness | $\eta$ $=\frac{I_{P}- E_{A}}{2}$ |
| The softness | $\sigma$ $=\frac{1}{\eta}$ |
| The electronegativity | $\chi=\frac{I_{P}+E_{A}}{2}$ |
| Electronic chemical potential | $\mu$ = - Electronegativity = $\mu= ̶$ |
| The Global electrophilicity index | ω $= \frac{\mu^{2}}{2\eta}$ |

**Table S2.** BET analysis of CELL-Cl and CELL@HBH.

| **Adsorbent** | **S_BET_(m²/g)** |
| --- | --- |
| **CELL-Cl** | 41.007 |
| **CELL@HBH** | 0.390 |

**Table S3.** The Global Reactivity Parameters determined using DFT/ B3LYP/6–31g (d, p) method of calculations for CELL, HBH, CELL@HBH, E122, E124, Cr(VI), CELL@HBH@E122, CELL@HBH@E124, CELL@HBH@Cr(VI).

| **Property** | **CELL** | **HBH** | **CELL@**  **HBH** | **Cr(VI)** | **E122** | **E124** | **CELL@HBH@E122** | **CELL@HBH @E124** | **CELL@HBH@Cr(VI)** |
| --- | --- | --- | --- | --- | --- | --- | --- | --- | --- |
| **E_HOMO_ [eV]** | **-0.2587** | **-0.1947** | **-0.1931** | **-0.2234** | **-0.1956** | **-0.1951** | **-0.1976** | **-0.2025** | **-0.2165** |
| **E_LUMO_ [eV]** | **0.04166** | **-0.0282** | **-0.0275** | **-0.0581** | **-0.081** | **-0.0761** | **-0.1138** | **-0.1036** | **-0.14997** |
| **Δ E_gap_ [eV]** | **0.3004** | **0.16643** | **0.16557** | **0.1653** | **0.11459** | **0.11909** | **0.08384** | **0.09892** | **0.06648** |
| **η [eV]** | **0.1502** | **0.083215** | **0.082785** | **0.08265** | **0.057295** | **0.059545** | **0.04192** | **0.04946** | **0.03324** |
| **σ [eV]^-1^** | **6.65779** | **12.01706** | **12.07948** | **12.09921** | **17.45353** | **16.79402** | **23.85496** | **20.21836** | **30.08424** |
| **I_p_ [eV]** | **0.25874** | **0.19466** | **0.19306** | **0.2234** | **0.19561** | **0.19514** | **0.19764** | **0.20248** | **0.21645** |
| **E_A_ [eV]** | **-0.04166** | **0.02823** | **0.02749** | **0.0581** | **0.08102** | **0.07605** | **0.1138** | **0.10356** | **0.14997** |
| **χ [eV]** | **0.10854** | **0.111445** | **0.110275** | **0.14075** | **0.138315** | **0.135595** | **0.15572** | **0.15302** | **0.18321** |
| **µ [eV]** | **-0.10854** | **-0.111445** | **-0.110275** | **-0.14075** | **-0.138315** | **-0.135595** | **-0.15572** | **-0.15302** | **-0.18321** |
| **ω [eV]** | **0.039217** | **0.074626** | **0.073447** | **0.119846** | **0.166952** | **0.154387** | **0.289226** | **0.236708** | **0.504902** |
| **The dipole moment (μ) [Debye]** | **1.872** | **2.3422** | **3.4722** | **6.2726** | **4.3449** | **7.7487** | **8.8176** | **8.7955** | **11.5333** |

**Table S4.** Langmuir, Freundlich, and Dubinin–Radushkevich isotherm constants and correlation coefficients of CELL@HBH for the three pollutants.

| **Langmuir isotherm constants** | | | | | | | |
| --- | --- | --- | --- | --- | --- | --- | --- |
|  | **K_L_(L/g)** | **q_m_ (mg/g)** | **R^2^** | **R_L_** | **𝝌^2^** | **MSE** | **SSE** |
| **E122** | 5.28 | 462.96 | 0.999 | 7.567 x 10^-4^ | 2.449 | 189.035 | 1134.210 |
| **E124** | 19.33 | 338.98 | 1.000 | 3.45 x 10^-4^ | 5.38 x 10^-4^ | 0.0364 | 0.182 |
| **Cr(VI)** | 0.12 | 306.75 | 0.996 | 7.9 x 10^-2^ | 221.897 | 13613.29 | 68066 |
| **Freundlich isotherm constants** | | | | | | | |
|  | **K_F_** | **N** | **R^2^** | | **𝝌^2^** | **MSE** | **SSE** |
| **E122** | 292.382 | 15.82 | 0.007 | | 84.357 | 5645.869 | 33875.216 |
| **E124** | 302.478 | 38.51 | 0.968 | | 5.79 x 10^-4^ | 0.0392 | 0.196 |
| **Cr(VI)** | 296.689 | 87.34 | 0.049 | | 244.558 | 18429.89 | 192080.78 |
| **D–R isotherm constants** | | | | | | | |
|  | **K** | **E (kJ/mole)** | | **q_m_ (mg/g)** | | **R^2^** | |
| **E122** | 5.898 x 10^-9^ | 9.207 | | 505.223 | | 0.969 | |
| **E124** | 6.262 x 10^-9^ | 8.935 | | 340.018 | | 0.997 | |
| **Cr(VI)** | 5.502 x 10^-9^ | 9.532 | | 218.547 | | 0.958 | |

**Table S5.** Analytical results of adsorption of E122, E124 and Cr(VI) in natural water samples employing CELL@HBH as an adsorbent. (n=3).

| **Sample** | **Pollutant** | **Spiked (µg/mL)** | **Measured**  **(µg/mL)** | **Recovered (µg/mL)** | **Recovery (%)** | **RSD (%)** |
| --- | --- | --- | --- | --- | --- | --- |
| **Tap Water** | **E122** | 0.00 | 0.00 | 0.00 | 0.00 | 0.00 |
|  |  | 50 | 0.105 | 49.895 | 99.790 | 1.44 |
|  |  | 100 | 0.506 | 99.494 | 99.494 | 1.53 |
|  |  | 150 | 0.701 | 149.299 | 99.533 | 1.18 |
|  |  | 200 | 9.379 | 190.621 | 106.631 | 1.39 |
|  | **E124** | 0.00 | 0.00 | 0.00 | 0.00 | 0.00 |
|  |  | 50 | 0.125 | 49.875 | 99.750 | 1.29 |
|  |  | 100 | 0.201 | 99.799 | 99.799 | 1.23 |
|  |  | 150 | 0.00 | 150 | 118.101 | 1.11 |
|  | **Cr(VI)** | 0.00 | 0.00 | 0.00 | 0.00 | 0.00 |
|  |  | 50 | 0.989 | 49.011 | 98.022 | 1.19 |
|  |  | 100 | 27.269 | 72.731 | 99.275 | 1.30 |
| **Waste water** | **E122** | 0.00 | 0.00 | 0.00 | 0.00 | 0.00 |
|  |  | 50 | 0.611 | 49.389 | 98.778 | 1.15 |
|  |  | 100 | 1.011 | 98.989 | 98.989 | 1.25 |
|  |  | 150 | 1.329 | 148.671 | 99.114 | 1.51 |
|  |  | 200 | 23.158 | 176.842 | 98.924 | 1.22 |
|  | **E124** | 0.00 | 0.00 | 0.00 | 0.00 | 0.00 |
|  |  | 50 | 0.005 | 49.995 | 99.990 | 0.95 |
|  |  | 100 | 0.099 | 99.901 | 99.901 | 1.12 |
|  |  | 150 | 0.669 | 149.311 | 117.575 | 1.21 |
|  | **Cr(VI)** | 0.00 | 0.00 | 0.00 | 0.00 | 0.00 |
|  |  | 50 | 1.105 | 48.895 | 97.790 | 1.32 |
|  |  | 100 | 28.368 | 71.632 | 97.775 | 1.26 |
| **Sea Water** | **E122** | 0.00 | 0.00 | 0.00 | 0.00 | 0.00 |
|  |  | 50 | 0.139 | 49.861 | 99.722 | 1.48 |
|  |  | 100 | 0.251 | 99.749 | 99.749 | 1.41 |
|  |  | 150 | 31.526 | 118.474 | 78.983 | 1.35 |
|  |  | 200 | 80.366 | 119.634 | 70.645 | 1.27 |
|  | **E124** | 0.00 | 0.00 | 0.00 | 0.00 | 0.00 |
|  |  | 50 | 4.197 | 45.803 | 91.606 | 0.99 |
|  |  | 100 | 18.522 | 81.478 | 81.478 | 1.10 |
|  |  | 150 | 68.953 | 81.047 | 63.811 | 1.20 |
|  | **Cr(VI)** | 0.00 | 0.00 | 0.00 | 0.00 | 0.00 |
|  |  | 50 | 3.781 | 46.219 | 92.438 | 0.97 |
|  |  | 100 | 46.368 | 53.632 | 73.205 | 1.17 |
